# FIMOFs: Fiber-Integrated Metal–Organic Frameworks Through Electrospinning

**DOI:** 10.3390/polym17081106

**Published:** 2025-04-19

**Authors:** Mine G. Ucak-Astarlioglu, P. U. Ashvin Iresh Fernando, Spencer A. Spane, Sulymar A. Rodriguez, Gilbert K. Kosgei, Charles A. Weiss, Ivan P. Beckman, Byron Villacorta, Sasan Nouranian, Ahmed Al-Ostaz

**Affiliations:** 1 U.S. Army Engineer Research and Development Center, Geotechnical and Structures Laboratory, 3909 Halls Ferry Road, Vicksburg, MS 39180, USA; spence1010@aol.com (S.A.S.); charles.a.weiss@erdc.dren.mil (C.A.W.J.); 2 SIMETRI, Inc., Winter Park, FL 32792, USA; ashvinfernando8@gmail.com; 3 U.S. Army Engineer Research and Development Center, Cold Regions Research and Engineering Laboratory, 72 Lyme Rd, Hanover, NH 03755, USA; ivan.p.beckman@usace.army.mil; 4 U.S. Army Engineer Research and Development Center, Environmental Laboratory, 3909 Halls Ferry Road, Vicksburg, MS 39180, USA; sulymar.a.rodriguez@usace.army.mil (S.A.R.); gilbert.k.kosgei@usace.army.mil (G.K.K.); 5 Department of Chemical Engineering, University of Mississippi, Oxford, MS 38677, USA; bsvillac@olemiss.edu (B.V.); sasan@olemiss.edu (S.N.); 6 Center for Graphene Research and Innovation, University of Mississippi, Oxford, MS 38677, USA; alostaz@olemiss.edu; 7 Department of Civil Engineering, University of Mississippi, Oxford, MS 38677, USA

**Keywords:** electrospinning, metal–organic frameworks (MOFs), fiber, composites, polymer composites, porosity, surface area, green, sustainable, multifunctional, military, civilian, adsorption environment

## Abstract

Green synthesis plays a crucial role in advancing sustainability within materials science. This study explores the integration of metal–organic frameworks (MOFs), obtained through green synthesis, using an electrospinning post-processing technique to develop MOF-based composite materials. The resulting novel multifunctional composites demonstrate enhanced stability and functionality, compared to their control counterparts. The integration of four types of MOFs into an electrospun fiber network was investigated using a specific polymer solution. Characterization and preliminary adsorption studies were conducted to elucidate the chemistry, morphology, and adsorptive capabilities of the resulting MOF composites. Electrospinning MOFs into polymer fibers improved their stability and dye removal capabilities. More specifically, optimization of MOF-to-polymer ratios and processing conditions yielded composites that are thermally stable, with modified surface area and porosity. Post-processing MOFs resulted in a fiber diameter increase of 44 and 109%, enhancing the composites by providing more MOF active sites and improved mechanical strength. Zirconium-based post-processed MOFs demonstrated superior dye removal, different from the copper-based dyes. Electrospinning technology has demonstrated significant potential in the fabrication of high-performance multifunctional MOF composites. This has helped to create advanced sustainable composites with tailored properties, paving the way for more targeted and efficient applications. The applications of these composites show promise for military engineering where durable, light weight, and multifunctional materials are critical in contributing to improved performance, operational efficiency, and safety.

## 1. Introduction

Metal–organic frameworks (MOFs) are crystalline compounds consisting of metal ions or clusters connected by organic ligands to form one-, two-, or three-dimensional structures. MOFs have large surface areas, high porosities, tunable chemical structures, and high stability [1]. MOF properties can be significantly enhanced through their composite formation [2,3,4]. MOFs’ unique three-dimensional porous structure and semiconductor-like properties inhibit the recombination of electron–hole pairs and facilitate them as charge carriers, making them promising candidates towards photocatalytic applications and energy harvesting [5,6]. MOFs improve composite materials’ performance and stability [7,8,9] MOFs’ tunable structure and compositional diversity with their unsaturated metal site coordination and/or special ligand functional groups, secondary building units (SBUs), make them suitable for heterogeneous applications [10,11] and for environmental biosystems [12].

MOF post-processing presents a unique opportunity for better amalgamated and stabilized fibers with enhanced functionality [13] and for tailoring their unique functionality, stability, porosity, and morphology for targeted applications. In this work, we have used electrospinning (ES) as a post-processing technique for this purpose.

MOF-based polymer nanofiber composites can be post-processed using an ES technique based on an electrostatic force drawing process by high-voltage application. In this process, a liquid droplet is electrified to generate a jet that is followed by stretching and elongation of fibers [14]. The evaporation of the solution results in the formation of a nanofiber [15,16] which is collected on a roller or a platform, fabricating samples in the forms of powders, fibers, and thin films [17,18]. This study aims to use the ES to affix the MOFs (via non-covalent-based interactions) onto nanofibers, creating a membrane with a fluffy porous structure.

Morphologies of MOF-integrated polymer electrospun fiber networks strongly depend on ES parameters, solution, and the environment, which mainly include temperature and humidity. Qin et al. have shown that ES produces flexible, MOF-based nanofiber structures that are self-supportive with high porosity and can be used in passive indoor moisture control [19]. Fixation of such MOF particles on the nanofibers results in the formation of thin non-woven membranes.

In fabricating MOF-based-nanofiber composites, (a) MOF-in-fiber, (b) MOF-on-fiber, and (c) MOF–seed–fiber methods are widely used [20,21]. In the MOF-in-fiber method, pre-synthesized MOF powder and polymers are mixed into the spinning solution at the same time and under high voltage, where the fibers wrap the MOF directly for strengthened stability. In the MOF-on-fiber method, MOFs are synthesized and deposited on the surface of blank fibers, which makes MOF particles attached to the fiber surfaces become more exposed sites with possible leaks due to the lack of chemical interactions [4]. In the MOF–seed–fiber, via in situ growth, MOF particles penetrate the fiber and grow inside and at the surface of the fiber, where one of the precursors of MOF is blended with polymers and spun into fiber by ES followed by immersion into a metal solution [22]. This enables better control of MOFs as they are pre-synthesized and characterized before ES.

In this study, we have reported the synthesis, ES process, and characterization of four different MOFs, which are two zirconium-based MOFs, UIO-66 (UIO denotes Oslo University, where it was first reported) and MIP-202, two were copper-based (Cu_3_(BTC)_2_(H_2_O)_3_) HKUST-1 MOFs, and one was a bio-based BIO-MOF-1 [23,24,25,26]. These MOFs were selected due to their distinct structural features including aromaticity, types of linkages, type of secondary building unit (SBU), and their extensive representation in the literature. These differences are critical because variations in aromaticity can be related to electron delocalization and chemical stability, while diverse linkage types influence the framework connectivity and functional performance. Out of these four MOFs synthesized, MIP-202 and BIO-MOF-1have not been reported in the literature for post-processing by the ES technique. Established presence of UIO-66 and HKUST-1MOFs in prior studies not only validates their relevance but also facilitates a direct comparison of our findings with existing data. UIO 66 and HKUST-1 have previously been post-processed by an electrospinning technique and used in several applications such as energy storage and environmental applications [11,27,28].

Scalable methods for fabricating sustainable, high-performance multifunctional nanocomposites are essential for military and civilian applications. This study explores the use of electrospinning as a post-processing technique in the development of MOF-based composite materials. The fabricated nanocomposites are characterized and tested for a specific application, demonstrating how the post-processing approach enables tailored material properties for a targeted application.

## 2. Materials and Methods

All materials and chemicals used in this study are detailed in the Supplementary Information document with the synthesis of all four types of MOFs. Initially several ES parameters such as polymer concentration, flow rate, and electrical voltage were studied and optimized in the presence of MOFs as shown in Table S1. Optimized ES conditions yield to the most homogeneous electrospun mats with a reasonable yield. The electrospinner, stirred sample ES solution, HKUST-1 in PAN, and the electrospun mat collected on mesh roller, and collection during the ES process are shown in Figure S1. Detailed information on ES parameter adjustment and optimization is also included in the Supplementary Information. Figure S2 shows the chemical structure of the PAN polymer, MOF-PAN solution sprayed from the 18-gauge needle, and XRD sample holders with electrospun MOF-PAN mat. Figures S3 and S5 presents the synthetic schemes for MIP-202 and UIO-66 synthesis, respectively. The physical appearances of synthesized MIP-202, UIO-66, HKUST-1, and BIO-MOF-1are given in Figure S4, Figure S6, Figure S7 and Figure S8, respectively. Subsequently, chemical, thermal, and morphological properties of electrospun MOFs were analyzed. Figure S9 presents the pictures of HKUST-1, MIP-202, UIO-66, and BIO-MOF-1samples before and after electrospinning. Data related to SEM and SEM-EDX measurements, and average elemental composition of electrospun MOFs are presented in Figures S10–S21, and Table S2. BET and DFT based surface area, pore volume, and pore diameter measurements are shown in Table S3. Fiber diameter measurement through SEM EDX with contours provided are shown in Figure S22. Finally, dye adsorption study was conducted using electrospun MOFs as the adsorbent as presented in Figures S23–S26 and Table S4.

## 3. Results and Discussion

**Figure 1:**
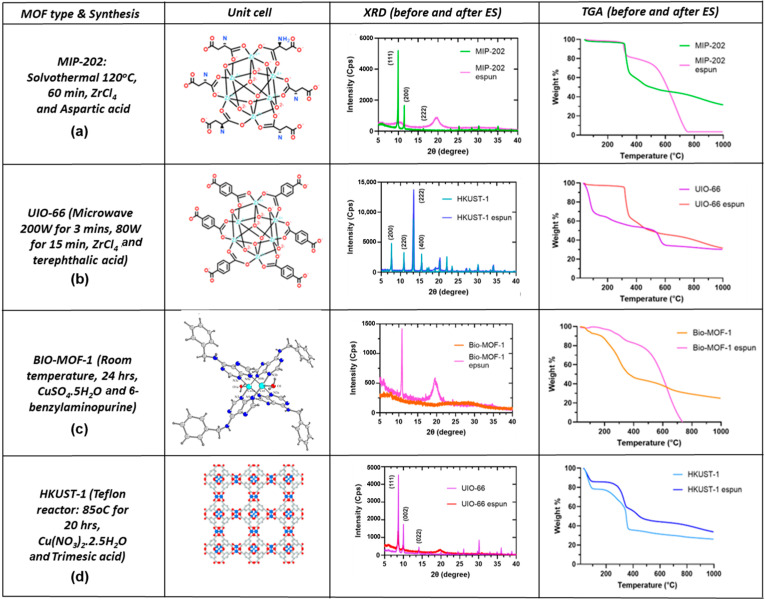
Unit cell crystal structures of MOFs, reaction conditions, and the metals, and ligands (SBUs) used for their synthesis. PXRD patterns and TGA analyses of UP-MOFs and their electrospun–MOF composites: (**a**) MIP 202 and (**b**) UIO-66. Images obtained from the CD Bioparticles Drug Delivery Catalog Nos CDM-CH246 [30] and CDM-ST008 [32], respectively. (**c**) Bio MOF-1 reprinted with permission from Yang et al. [26], and (**d**) Illustration of HKUST-1, reprinted with permission from Kim, H. K. [29].

Four types of MOFs—HKUST-1 [29], Bio MOF-1 [26], MIP 202 [30,31] and UIO-66 [32] investigated in this research are shown in Figure 1 along with their X-ray diffraction (XRD) and thermogravimetric analysis (TGA) characterizations. Synthetic schemes of UIO-66 and MIP-202, and physical appearances all MOFs are detailed in Figures S2–S7. In the synthesis of BIO-MOF-1and MIP-202, green solvents were used with sustainable linkers (Bio-MOF-1: 6-benzylaminopurine and MIP-202: L-aspartic acid) for potential green applications. MIP-202 and BIO-MOF-1were chosen due to their suitability for ES post-processing, indicating their potential use in military applications. UIO-66 and HKUST-1 MOFs were chosen due to their wide range of applications, serving as benchmark structures from hydrogen storage [33] to separation, and electrocatalysis [34]. Our previous work with MIP-202 has shown promise in hydrolyzing the nerve agent surrogate DFP [35]. Each MOF provides a unique structural morphology and related functionality.

XRD, TGA, and surface area and pore size analyses reveal that all four MOFs exhibit distinct differences in their chemistry, pore size distribution, surface area, and crystallographic structure offering a wider range of applications after post-processing through ES. Crystallographic analyses show that MIP-202 [30] has a cubic arrangement and UIO-66 [32] a face-centered cubic (FCC) arrangement with an octahedral morphology; HKUST-1 has paddlewheel-type clusters, while BIO-MOF-1has crystals that occupy the Hoogstenn face, as presented in Figure 1.

Averaged particle sizes of the MOFs studied varied and were as follows: UIO-66 400–500 nm [36], HKUST-1 2-6 µm [37], MIP-202 95–650 nm [38], and BIO-MOF-1170–240 nm [26]. These structural differences, along with their porosity and chemical functionality, allow MOF composites to be used in several applications, including photochemical sensing, toxicant capture, and CO_2_ capture. Variations in crystallographic structures and particle sizes are expected to affect the post-processing of MOFs and require ES parameter optimization for each individual MOF. This work will showcase whether MOFs can be post-processed using an ES technique and optimized based on the type of MOF of interest.

A 9% *w*/*v* polyacrylonitrile (PAN) polymer was chosen due to its smooth, homogeneous, and consistent electrospun mat fabrication compared to other PAN-based compositions. Previous studies demonstrated that PAN, when mixed with other materials such as carbon nanofibers, cellulose, and lignin resulted in a homogeneous electrospun fiber network [39,40] and as a result, PAN was selected for this study. Dissolving PAN in dimethylformamide (DMF), and mixing it with MOFs, MOF-polymer matrix was formed and electrospun mat obtained as shown in Figure S1, and Tables S1 and S2. After trials of various amounts of MOFs in PAN, an optimum ES solution combination of 9% *w*/*v* PAN and 10% *w*/*v* MOF was found based on the analysis of SEM/EDS analysis and consistent flow of solution. A probe sonicator was used in the preparation of the homogeneous MOF-PAN ES solution. An 18-gauge needle provided the most flawless electrospun mats, different from the ones obtained with smaller gauge needles. At an electrical charge of 18–28 kV, the ES solution was pushed through the needle. The ES solution droplets at the tip of the needle were then sprayed onto a roller, which resulted in a fibrous mat. Throughout this study, 9% *w*/*v* PAN and 10% *w*/*v* MOF solution was used in fabricating all electrospun mats. Upon fabrication, the fibrous mats were subjected to heating at 60 °C to remove the residual solvent. Ongoing research focuses on the carbonization of these MOF-based-PAN composite fibers.

This study successfully presented the effective integration of diverse MOFs into electrospun polymer fibers and revealed significant changes in the structural, thermal, and adsorptive properties of electrospun MOFs.

### 3.1. Unprocessed MOFs (UP-MOFs), Post-Processed MOFs (PP-MOFs), and Structural Modification due to ES Process

The electrospinning process proved to be an invaluable tool for fabricating and tailoring PP-MOF polymer composites. Characterization techniques revealed important structural and chemical properties of the UP- and PP-MOFs in understanding their behavior and potential applications. Figure 1 shows the X-ray diffraction patterns illustrating the impact of the ES on MOF crystallinity showing the structure-dependent response. UP-MOFs showed an agreement with the literature. UP-HKUST-1 MOFs shows diffraction patterns that are related to 200, 220, 222, and 400 planes [41,42]. UP-UIO-66 shows all three major XRD peak pattern characteristics at 111, 002, and 022 [43,44]. UP- and PP-MIP-202 also match well with previous work [35]. HKUST-1 retained its characteristic peaks after pro-processing due to its tri-functional benzene ring making its structure more rigid than the others, showcasing its robustness during the ES process, with no significant change in its crystalline phases. In contrast, UIO-66 and MIP-202 were characterized by more flexible, di-functional and non-aromatic linkers, respectively. MIP-202 was the only MOF studied in this research with a flexible SBU and no aromaticity. As a result of these structural characteristics, UIP-66 and MIP-202 showed a notable loss in crystallinity, with the disappearance of most of their XRD patterns and the broadening of the peak at 2θ = 20° indicating a significant disruption in their structure. The XRD of PP-MIP-202 exhibited a significant reduction in peak intensity for angles above 2θ = 23°, as observed in UiO-66. PP-UIO-66 presented a pristine MOF by matching with the XRD pattern in the literature [45]. It was reported that when UIO-66 is embedded within a polymer structure, some of its lower-intensity XRD peaks disappear. Remarkably, BIO-MOF-1, which was initially amorphous, transitioned to a semi-crystalline state by undergoing a polymer-induced structural ordering with the emergence of a sharp peak at 2θ = 11° and another broad peak at 2θ = 20°. This suggests that amorphous MOFs, such as Bio-MOF-1, can form strong interactions with the cyano groups that are present in PAN, bringing semi-crystallinity to the post-processed MOF structure. The presence of these interactions could result in the formation of more rigid and stable structures, which would be beneficial for the performance of the material.

### 3.2. Thermal Stability

Figure 1 presents the thermogravimetric analysis (TGA) plots highlighting remarkable improvements in the thermal stability of PP-MOFs. These enhancements are characterized by a delayed onset of decomposition and reduced weight loss, distinguishing them from the patterns observed in the UP-MOFs. This improvement in thermal stability may be attributed to the enhanced structural integrity of MOFs achieved through ES post-processing. For example, PP-HKUST-1 showed a 45% reduction in weight loss, compared to 50% for UP-HKUST-1, indicating a slower decomposition of benzene-1,3,5-tricarboxylate linkers. PP-UIO-66 showed a delayed onset compared to UP-UIO-66, with a reduced weight loss of 35% compared to 40% for UP-UIO-66 due to the decomposition of terephthalate linkers, suggesting better thermal stability due to fewer defects and improved crystallinity. Similarly, BIO-MOF-1 exhibited a gradual weight loss of 40%, in contrast to the 45% loss observed in its unprocessed counterpart. This difference attributed to the breakdown of organic linkers at the major decomposition phase, that occurred with a delayed decomposition, suggesting enhanced structural integrity. Lastly, electrospun MIP-202 shows slightly improved thermal stability with a higher decomposition onset temperature and a reduced rate of weight loss. These observations suggest that ES enhances structural integrity, reduces defects, and potentially alters surface area and porosity, contributing to the improved thermal profiles of MOFs and making electrospinning a promising technique for expanding MOFs applications in high-temperature environments. The amount of material remained after TGA analyses is reported as the char yield in Table S4.

### 3.3. Fiber Morphology, BET Surface Area, and Pore Size Distribution

**Figure 2:**
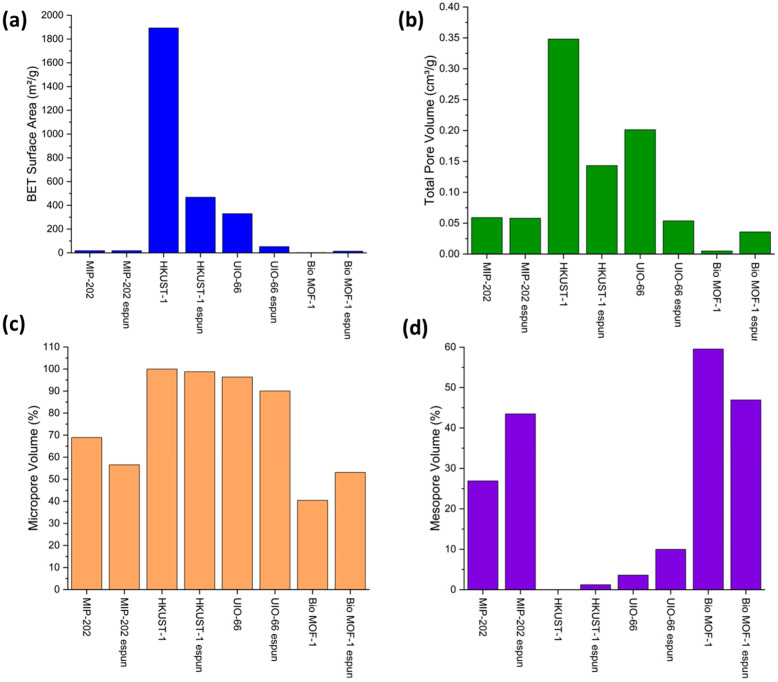
Surface area and pore volume characterization of UP-MOFs and PP-MOFs. (**a**) Multi-point BET-based surface area; (**b**) total pore volume [calculated via DFT method]; (**c**) percentage micropore volume [calculated by sum of pore volume between 0 and 9.7 nm/sum of total pore volume × 100]; and (**d**) percentage mesopore volume [calculated by sum of pore volume between 10 and 40 nm/sum of total pore volume × 100].

Analyses of MOFs’ surface area and porosity indicated the impact of ES post-processing on their properties. Figure 2a,b presents the Brunauer–Emmett–Teller (BET) and Density Functional Theory (DFT) analyses for MOFs’ surface area and pore volume studies after ES post-processing, respectively. Based on the analyses, PP-MOFs exhibit a general trend of a reduced surface area and pore volume, possibly due to framework collapse and/or restructuring upon voltage application of ES and partial blockage by the polymer matrix. However, UP-BIO-MOF-1 showed a high surface area and pore volume, attributed due to its benzylaminourine linker, indicating the importance of linker chemistry affecting material properties. Upon ES post-processing, HKUST-1’s surface area decreased significantly from 1892.97 m^2^/g to 468.44 m^2^/g, with a 75% reduction, and that of UIO-66 from 329.91 m^2^/g to 52.47 m^2^/g, with an 84% reduction. On the contrary, BIO-MOF-1 showed an increase from 2.28 m^2^/g to 13.08 m^2^/g, with a 513% increase. The micropore (0–9.7 nm) volume percentages were found to decrease for all PP-MOFs except for Bio MOF-1. The micropore volume percentage of MIP-202 decreased from 68.90% to 56.53%, with an 18% reduction, whereas that of BIO-MOF-1increased from 40.45% to 53.07%, with an increase of 31%; HKUST-1 changed from 99.98% to 98.76%, with a decrease of 1%, and UIO-66 from 96.37% to 90.03%, with a decrease of 7%.

**Figure 3:**
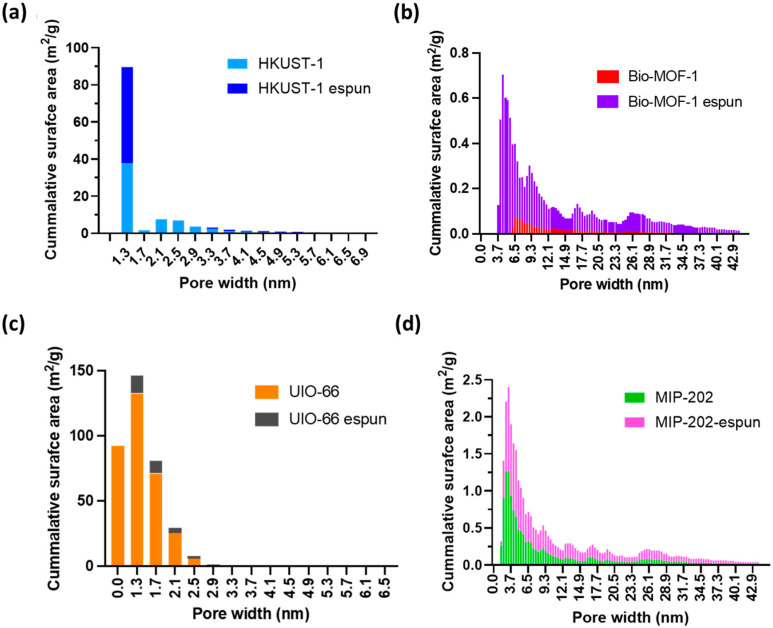
Cumulative surface area vs. pore width for UP-MOFs- and PP-MOFs (**a**) HKUST-1, (**b**) Bio-MOF-1, (**c**) UIO-66 and (**d**) MIP-202.

Figure 2c,d show an increase in porosity and transitioning from microporosity to mesoporosity as a result of ES post-processing that tailors pore size distribution. The reduction in micropore volume and increase in mesopore volume, except for Bio-MOF-1, may indicate merging of smaller pores or the formation of larger ones. This feature enables accessibility for specific target molecules as shown in Table S3. Figure 3 shows the cumulative surface area vs. pore width for both UP-MOFs and PP-MOFs, where all four UP-MOFs exhibit high crystallinity and tight structures with smaller pores and more compact frameworks. This generally results in a higher selectivity in adsorption but potentially a lower surface area and total pore volume. On the contrary, PP-MOFs show a broader pore size distribution, surface area, and pore volume, indicating the possibility of ES leading to structural changes that improve accessibility and overall composite performance, especially for applications requiring larger pore volumes and surface areas. PP-UIO 66 shows minimal change from UP-UIO-66, indicating the possibility of staying structurally intact during the ES process, different from the other MOFs analyzed.

Figure 3 presents the cumulative surface areas vs pore widths (nm) for unprocessed and electrospun MOFs. This figure indicates that all four UP-MOFs exhibit highly crystalline and tight structures with smaller pores and more compact frameworks. This generally results in higher selectivity in adsorption but potentially lower surface areas and total pore volumes. On the contrary, electrospun MOFs show a broader distribution in pore size, surface area, and pore volume, indicating that the ES may lead to structural changes that improve accessibility and overall performance, especially in applications requiring larger pore volumes and surface areas. UP-UIO 66 shows minimal change upon ES post-processing suggesting that UIO-66 stays intact during the ES process compared to others. Table S3 shows the effect of ES on the micro- and mesopore volumes of the post-processed composites. Table S3 indicates a decrease in the total pore volume of HKUST-1 from 0.35 cm^3^/g to 0.14 cm^3^/g. A similar trend was observed for PP-UIO-66 from 0.20 cm^3^/g to 0.05 cm^3^/g. On the contrary, an increase in the total pore volume for PP-Bio MOF-1 from 0.01 cm^3^/g to 0.04 cm^3^/g. Meanwhile, no change was observed in PP-MIP-202 pore volume at 0.06 cm^3^/g. In terms of the mesopore (10–40 nm) volume percentages, they show an increase in all electrospun MOFs, except for PP-Bio MOF-1, which decreased from 59.55% to 46.93%, with a reduction of 21%. PP-BIO-MOF-1’s contrasting trend in decreasing mesopore volume compared to other PP-MOFs indicates the importance of chemical structure in the ES process. An increase in the mesopore volume accompanied by a decrease in micropore volume suggests a potential trade-off in the pore size distribution, where larger pores may enhance accessibility for larger molecules, at the expense of overall surface area for adsorption. The pore diameter remained relatively consistent across all UP- and PP-MOFs.

The notable increase in the surface area and pore volume of PP-BIO-MOF-1highlights the unique role of linkers, in this case, the benzylaminopurine linker in facilitating favorable interactions within the PAN polymer. This observation suggests the importance of MOF linkers in tailoring porosity and performance enhancements in PP-MOFs.

### 3.4. Fiber Diameter Analysis, Scanning Electron Microscopy, and EDX Analysis

**Figure 4:**
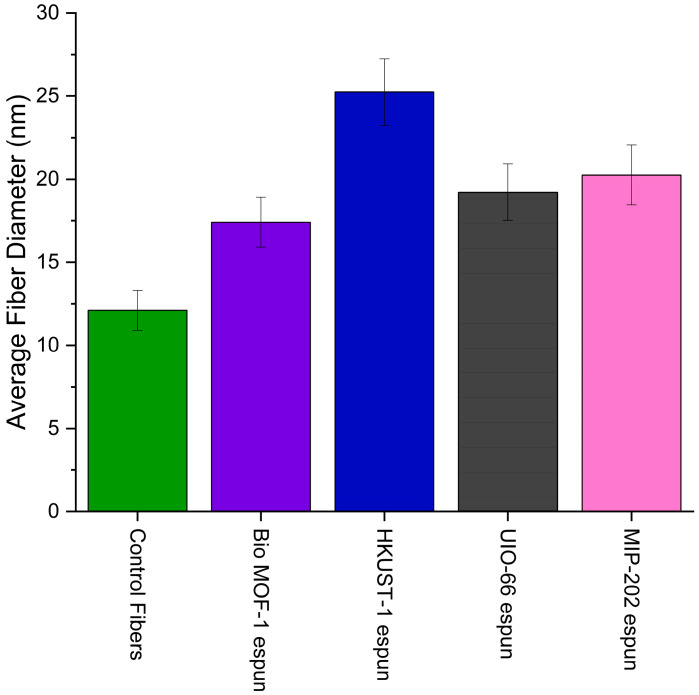
Average fiber diameters for control and PP-MOF composites.

**Figure 5:**
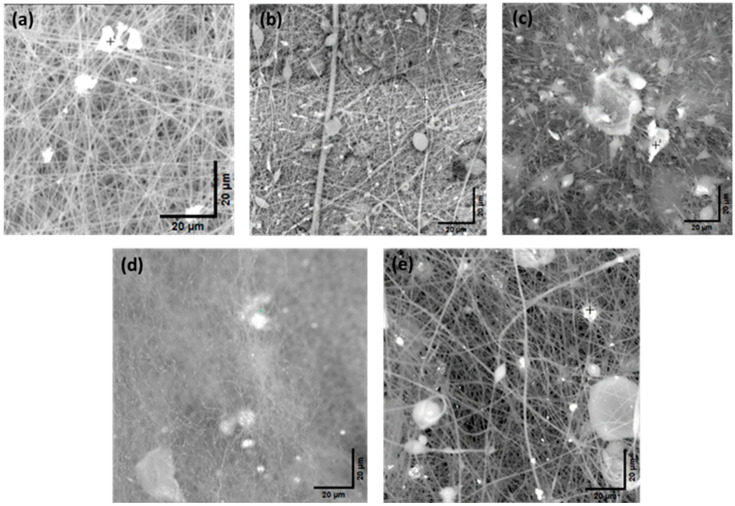
SEM images of (**a**) control, (**b**) PP-Bio-MOF-1, (**c**) PP-HKUST-1, (**d**) PP-UIO-66, and (**e**) PP-MIP-202 (scale bar at 20 µm).

Fiber diameter provides mechanical strength to structures and active sites for the interactions within the composite matrix. It also regulates the diffusion of molecules, optical properties, and thermal and electrical conductivity of the composites. Figure 4 shows the average fiber diameters for the control and PP-MOF composites and Figure 5 the energy-dispersive X-ray spectroscopy (EDX) analysis to gain insight into fiber morphology and metal distribution. The average diameters of PP-MOFs and their percentage increases compared to the control sample were as follows: PP-Bio-MOF-1, 17.40 µm and 43.7%; PP-HKUST-1, 25.24 µm and 108.5%; MIP-202, 20.25 µm and 67.3%; and UIO-66, 19.22 µm and 58.7%. The control samples without any MOFs shows the smallest average diameter among the samples studied, of 12.11 µm. The significant increase in the average diameters underscores the potential formation of larger fibers during the ES post-processing. These differences can be attributed to the diverse physical and chemical properties of UP-MOFs and their interaction with the polymer matrix during shaping the PP-MOF structure.

The morphologies of PP-MOFs were examined using SEM, while their metal content was assessed through EDX analyses. Figure 5 shows the SEM images of (a) control fibers (without MOFs), (b) Bio-MOF-1, (c) HKUST-1, (d) UIO-66, and (e) MIP-202. EDX analyses revealed a heterogeneous metal distribution in the PP-MOFs. The stoichiometric weight percentage of metals varied among the different MOFs, as revealed by spot and full-region scans. BIO-MOF-1spot scans in Figure S11 shows a metal content of 17.08% across three spots, while full-region scans show significantly lower content of 3.05%. On the other hand, HKUST-1 in Figure S12 demonstrates a relatively higher metal content in spot scans, with 48.03% for region 1 and 23.64% for region 2 across three spots, while full-region scan reveals a total metal content of 25.60%. UIO-66, in Figure S14 shows a consistent metal distribution, with spot scans showing 17.75% across four spots and 19.26% full-region scans making its performance more predictable. MIP-202 in Figure S17, shows a slightly lower metal content in full-region scans at 6.84%, compared to spot scans 8.22% across five spots, suggesting a minor, relatively consistent heterogeneity in the metal distribution.

EDX spot and region scans revealed a direct correlation in the metal content and its distribution, and the variations in the MOF fiber diameter. Higher and more heterogeneous metal content resulted in larger, more variable fiber diameters, while consistent metal content yielded in uniform fiber formation. These findings highlight the importance of the characterization and control of metal distribution in tailoring fiber properties for specific applications. It is noted that better dispersion was achieved using probe sonication. Testing micro- and nano-sized MOFs will be central in the performance optimization of PP-MOF composites.

### 3.5. Application: Enhanced Adsorptive Capacity

**Figure 6:**
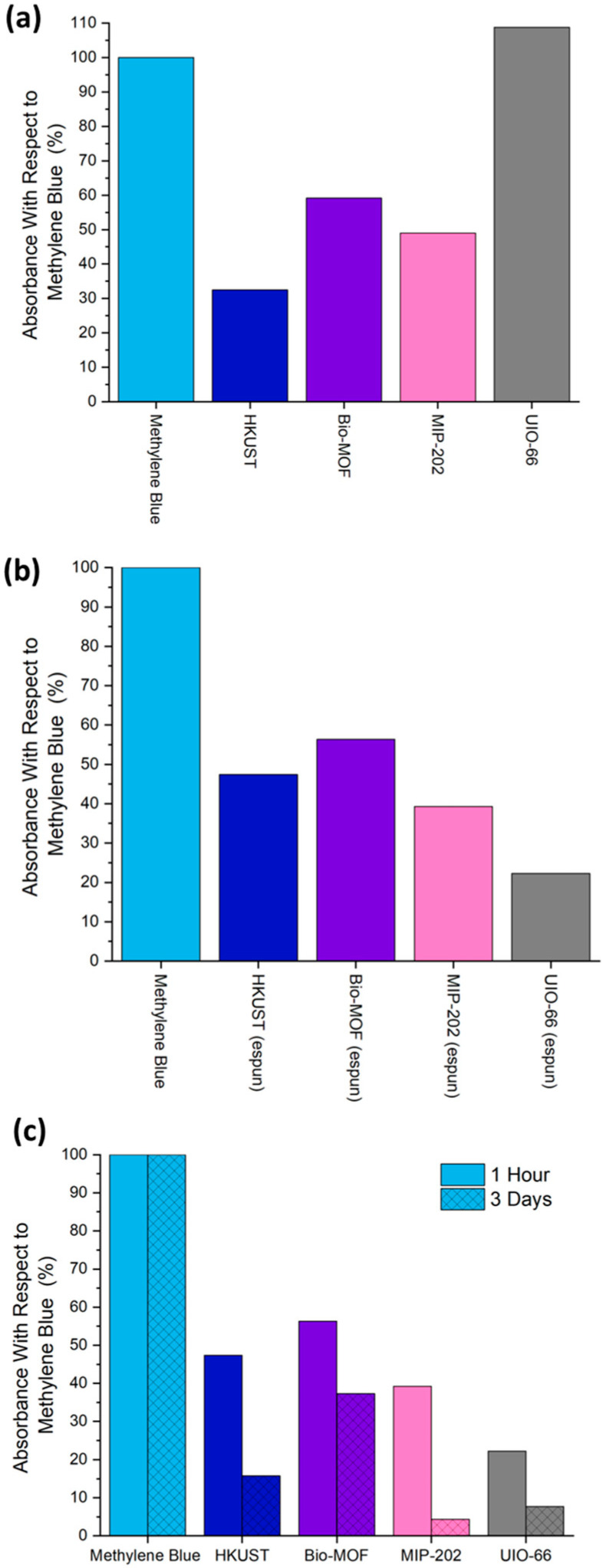
MOF adsorbent for effective MB dye removal: (**a**) UP-MOFs and (**b**) PP-MOFs at 1 h and (**c**) UV absorbance of MB in the presence of PP-MOFs at 1 h and 3 days.

Dye adsorption studies shown in Figure 6 and Figure S26 proved that PP-MOFs retained and, in some instances, enhanced their dye adsorption capabilities. The MB dye adsorption behavior of four PP-MOFs and their UP-MOFs was studied by subjecting all materials to MB exposure for 1 h and 3-day time intervals. The free MB dye exhibited 100% absorbance, serving as a baseline with no adsorption. UV-Vis absorption spectra confirmed that the MB dye removal was due to the adsorption of MB by the MOFs. The spectra revealed that the MOF–dye solutions consistently retained their major absorption maxima at approximately 680 nm, with no significant changes in the shape of the absorption curve. The only observable change was a reduction in peak height, indicating a decrease in dye concentration due to adsorption onto the MOFs. These findings confirmed that the dye molecules were being adsorbed onto the MOF surfaces without any chemical transformation, as shown in Figure S26. Time-based adsorption capability studies in Figure 6 show that MIP-202 and UIO-66 demonstrated the highest dye adsorption capacities, as evidenced by their low absorbance percentages among the composites. MIP-202 reduced dye absorbance to 39% after 1 h and furthered to 4% after 3 days, indicating significant and sustained dye adsorption. Similarly, UIO-66 reduced dye absorbance to 22% after 1 h and furthered to 8% after 3 days, showing strong adsorption capabilities. In contrast, HKUST-1 and BIO-MOF-1exhibited lower adsorption capacities. HKUST-1 reduced dye absorbance to 47% after 1 h and 16% after 3 days, while Bio-MOF showed an absorbance of 56% after 1 h and 37% after 3 days.

Upon ES post-processing, PP-MIP-202 and PP-UIO-66 exhibited substantial improvements in dye adsorption, with absorbance values decreasing to 39% and 22%, respectively. This suggested enhanced dye–MOF interaction within the fiber matrix. UIO-66 showed the most remarkable improvement in adsorption due to ES post-processing, which may be attributed to the increased dye accessibility and stabilization of active sites on the PP-UIO-66. BIO-MOF-1showed a slight improvement in adsorption, with absorbance decreasing from 59% to 56%, indicating a relatively stable performance due to ES post-processing. In contrast, HKUST-1 exhibited a decrease in adsorption performance, with absorbance increasing from 32% to 47%, possibly due to a reduced accessibility of active sites within the fiber matrix. Zirconium-based UIO-66 and MIP-202 demonstrated superior dye removal, different from copper-based MOFs such as HKUST-1 and Bio-MOF-1. Zirconium ions typically form robust and highly stable coordination bonds with negatively charged ligands or polar molecules due to their high charge density and ability to form strong metal-ligand interactions. In this case, the zirconium-based MOFs may provide more energetically favorable sites for the adsorption of cationic dye molecules like MB, enhancing their dye removal capacity. The superior hydrolytic stability of zirconium-based MOFs such as MIP-202 and UIO-66 could plays a critical role in maintaining their structural integrity and active sites during prolonged dye exposure in aqueous environments. In contrast, copper-based MOFs like HKUST-1 and Bio-MOF are more prone to partial degradation or loss of active sites under similar conditions, which could limit their adsorption efficiency over time. The framework rigidity and pore accessibility of zirconium-based MOFs might also provide better dye penetration and retention, further explaining their superior performance.

These adsorption studies demonstrated that PP-MOFs, specifically ones based on zirconium, are efficient in dye removal from aqueous solution applications. The MOFs’ optimal weight was found to be crucial in maximizing adsorption capacity, based on a MOF loading study on dye adsorption capacity (Figure S24). Adsorption was relatively high at 5 and 10 mg of HKUST-1; however, increasing the MOF amount beyond 10 mg did not enhance adsorption and instead showed a degree of desorption, suggesting that excessive MOF mass may lead to an aggregation or reduced accessibility of active sites. This observation highlights the importance of optimizing the amount of adsorbent for effective dye removal, where a 10% *w*/*v* MOF loading (10 mg of MOFs in 100 mL of polymer solution) was selected as the optimal formulation for PP-MOF composites. The results indicate the effect of ES post-processing on the MOFs in addition to the type and loading of MOFs used, along with their relatively different particle size (Figure S22) and shape, as shown in SEM images (Figures S18–S22) and chemical integration within the polymer matrix impacting the adsorption capacity of these materials. Zirconium-based MOFs that were ES-post-processed demonstrated stabilized active sites and great potential as effective adsorbent materials for dye adsorption from aqueous environments. In the future, adsorption kinetics and isotherm models may be employed for a deeper understanding of the adsorption mechanism and the interactions between zirconium-based MOFs and cationic dyes to further optimize the performance of these materials.

## 4. Conclusions

This research study has successfully demonstrated organic synthesis of MOFs, their fabrication into electrospun fibers, and their characterization, along with an application methodology for the successful removal of dyes from aqueous solutions and insights into the structural, thermal, and adsorption properties of these composites. The electrospinning method has proven to be a versatile approach for enhancing the stability and functionality of these composites, which open new avenues for targeted applications, particularly in environmental remediation. The fabricated MOFs provided thermal stability, controlled porosity, and improved adsorption capabilities for potential applications that can easily be tailored through scalable MOF synthesis and fabrication through electrospinning.

A PAN polymer (molecular weight 150,000 Da) was chosen due to its capability in the fabrication of a high-yield homogenous electrospun polymeric mat incorporating nanomaterials [44,45,46]. The cyano-functional groups of PAN make it an excellent choice in facilitating chemical interactions with the functional carboxylate groups of MOFs (MIP-202, HKUST-1, and UIO-66). The MOF-to-PAN ratio was optimized to 1.5:1 to improve the structural integrity of the electrospun mat. Additionally, the surface functionality of the UP-MOF improved the compatibility between the UP-MOF and the polymer matrix. A relatively high MOF amount was incorporated into the electrospun mat without compromising the structural integrity of the fibers. Characterization techniques provided valuable insights into the physical and chemical properties of the electrospun–MOF composites. XRD patterns indicated that while some UP-MOFs retained their crystalline structures after the ES process, others showed changes suggesting a partial loss of crystallinity or transformation into amorphous states. For example, electrospun HKUST-1 maintained its crystallinity, whereas UIO-66 and MIP-202 exhibited reduced peak intensities, indicating structural changes in ES post-electrospinning. SEM revealed that the incorporation of MOFs into fibers resulted in increased fiber diameters. EDX confirmed the heterogeneous distribution of metals within the fibers. Porosity and surface area analyses indicated that ES generally reduces the BET surface area and total pore volume of most of the MOFs, likely due to compression or structural changes during the ES process. For instance, the surface area of HKUST-1 decreased from 1892.97 m^2^/g to 468.44 m^2^/g, and for UIO-66, it was reduced from 329.91 m^2^/g to 52.47 m^2^/g. However, BIO-MOF-1showed an increase in surface area from 2.28 m^2^/g to 13.98 m^2^/g, suggesting unique interactions with the polymer matrix. It was observed that the smaller-surface-area MOFs showed better dispersion in the ES solution. Structural and adsorption changes occurred following ES post-processing, with zirconium-based MOFs demonstrating superior dye removal.

The scalability of MOFs and their integration into polymer matrices present opportunities for the future investigation. Maintaining uniformity and consistency in the fabricated PP-MOFs remains challenging and requires better control of electrospinning parameters, including flow rate, voltage, and humidity, and standardized protocols. Novel composites obtained through ES post-processing are expected to be promising, with unique properties from high mechanical strength to electrical and magnetic conductivity and adsorption; they can be used in a broader number of civilian and military applications.

In summary, this study has made significant advances in fabricating MOF-integrated electrospun polymer composites. The optimization of MOF quantities and the integration of multifunctional materials into polymer matrices have become straightforward processes. Future research should focus on optimizing the ES process for larger-scale production, improving reproducibility, and exploring new material combinations to unlock the full potential of these composites for various applications.

## Data Availability

The original contributions presented in this study are included in the article/Supplementary Information. Further inquiries can be directed to the corresponding author as needed.

## Supplementary Materials

The following supporting information can be downloaded at https://www.mdpi.com/article/10.3390/polym17081106/s1, Figure S1: (a) Electrospinner; (b) stirred HKUST-1 and PAN polymer dispersion; (c) and (d) collection of electrospun fiber mats during the electrospinning process; Figure S2: (a) Chemical structure; (b) MOF-PAN solution sprayed from the 18-gauge needle during the electrospinning process; (c) MOF-PAN electrospun mats placed onto XRD sample holders; Figure S3: Synthetic scheme for the synthesis of MIP-202; Figure S4: Physical appearance of MIP-202; Figure S5: Synthetic scheme for synthesis of UIO-66 (unit cell image of UIO-66 was obtained from www.cd-bioparticles.net/p/10359/uio-66, accessed on 31 March 2025); Figure S6: Physical appearance of UIO-66; Figure S7: Physical appearance and synthetic method of HKUST-1; Figure S8: Physical appearance of BiO-MOF-1; Figure S9: Pictures of HKUST-1, MIP-202, UIO-66, and BIO-MOF-1samples before and after electrospinning; Figure S10: Electrospinning BioMOF-1 at 10% *w*/*w* with respect to polymer mass—SEM EDX; Figure S11: Electrospinning BioMOF-1 at 10% *w*/*w* with respect to polymer mass—SEM EDX (combined elemental mapping); Figure S12: Electrospinning HKUST-1 at 10% *w*/*w* with respect to polymer mass—SEM EDX; Figure S13: Electrospinning HKUST-1 at 10% *w*/*w* with respect to polymer mass—SEM EDX; Figure S14: Electrospinning UiO-66 at 10% *w*/*w* with respect to polymer mass—SEM EDX; Figure S15: Electrospinning UIO-66 at 10% *w*/*w* with respect to polymer mass—SEM EDX; Figure S16: Electrospinning MIP-202 stirred for 1 h at 10% *w*/*w* with respect to polymer mass—SEM EDX; Figure S17: Electrospinning MIP-202 stirred for 1 h at 10% *w*/*w* with respect to polymer mass—SEM EDX; Figure S18: SEM images of HKUST-1 at (a) 890× and (b) 4800× magnification; Figure S19: SEM images of Bio-MOF- 1 at (a) 870× and (b) 5300× magnification; Figure S20: SEM images of UIO-66 at (a) 3800× and (b) 1650× magnification; Figure S21: SEM images of UIO-66 at (a) 1900× and (b) 7300× magnification; Figure S22: SEM EDX with contours; Figure S23: UV-Vis absorption spectra for the dye adsorption of FIMOFs at 1 h; Figure S24: Change in the absorption maximum of MB dye at 665 nm with varying amounts of MOF: (a) data without UIO-66 and (b) data with UIO-66. * Plots are shown to present the relatively higher absorption of UIO-66, different than that of other MOFs; Figure S25: UV-Vis absorption spectra for FIMOFs after 3-day-long dye exposure; Figure S26: (a) Change in the intensity of MB dye at two absorption maxima, 315 nm (top line), and 611 nm (bottom line), with varying amounts of UP-HKUST-1 powder, and (b) change in the intensity of MB dye at 611 nm for UP-HKUST-1 and PP-HKUST-1 after 1 h exposure to the dye; Table S1: Electrospinning parameters for different MOF-PAN solutions; Table S2: Average elemental composition of MOF–electrospun composites, Table S3: BET- and DFT-based data fits for all materials related to N_2_ physisorption; Table S4: TGA of UP-MOFs and PP-MOFs (FIMOFs). All supplementary information references are cited in the main text [23,24,25,26,31,38].
