# Supplementary material for: FIMOFs: Fiber-Integrated Metal–Organic Frameworks Through Electrospinning
Source: Polymers (Basel). 2025 Apr 19;17(8):1106. doi: 10.3390/polym17081106 (PMC12030668; doi:10.3390/polym17081106)
Supplement: Supplementary file 1 [file polymers-17-01106-s001.zip › polymers-3494104-supplementary.pdf]

polymers MDPI

Supporting Information

FIMOFs: Fiber Integrated Metal-Organic Frameworks Through Electrospinning

Mine G. Ucak-Astarlioglu [1§*], P U Ashvin Iresh Fernando [2,4§], Spencer A. Spane [1], Sulymar A. Rodriguez [3], Gilbert K. Kosgei [3], Charles A. Weiss Jr. [1], Ivan P. Beckman [4], Byron Villacorta [5,6], Sasan Nouranian [5,6], Ahmed Al-Ostaz [6,7]

[1]U.S. Army Engineer Research and Development Center, Geotechnical and Structures Laboratory, 3909 Halls Ferry Road, Vicksburg, MS, 39180 USA
[2]SIMETRI, Inc. Winter Park, FL 32792 USA
[3]U.S. Army Engineer Research and Development Center, Environmental Laboratory, 3909 Halls Ferry Road, Vicksburg, MS, 39180 USA
[4]U.S. Army Engineer Research and Development Center, Cold Regions Research and Engineering Laboratory, 72 Lyme Rd, Hanover, NH 03755 USA
[5]Department of Chemical Engineering, University of Mississippi, University, MS 38677, USA
[6]Center for Graphene Research and Innovation, University of Mississippi, University, MS 38677, USA
[7]Department of Civil Engineering, University of Mississippi, University, MS 38677, USA

§ These authors contributed equally to this work.

*Corresponding Author:
Mine.G.Ucak-Astarlioglu@usace.army.mil

Materials and Methods

Chemicals used in this work

All chemicals in this study were purchased from Sigma Aldrich (MilliporeSigma, St. Louis, MO, USA) and used as is without further purification, unless otherwise noted. DMF (N,N-Dimethylformamide, CAS 68-12-2), $ZrCl_4$ (Zirconium (IV) chloride, CAS 10026-11-6), 1,4-benzenedicarboxylic acid (terephthalic acid, CAS 100-21-0), acetic acid (CAS 64-19-7), and DI water obtained from MilliQ system (Merck Millipore, Burlington, MA, USA). -1,3,5-tricarboxylic acid (trimesic acid, CAS 554-95-0) and Cu (II) nitrate hemipentahydrate (Copper (II) nitrate hemipentahydrate, CAS 19004-19-4), $CuSO_4{\cdot}5H_2O$ (Copper (II) sulfate pentahydrate, CAS 7758-99-8) and 6-benzylaminopurine (CAS 1214-39-7). For electrospinning, polyacrylonitrile (PAN, CAS 25014-41-9) was dissolved in DMF (CAS 68-12-2). Dye studies were conducted using Methylene Blue (CAS 7220-79-3).

Instrumentation

BET N_2 physisorption: The physisorption studies were performed using an Anton-Parr Nova 600 BET system (Anton Paar, Ashland, VA, USA). A 100-150 mg sample was used for testing. Solid samples were placed into a glass type B long cell with a bulb (internal diameter 6 mm), weighed and subjected to degasification (at 80 ºC for 4 hrs at high vacuum to minimize framework breakdown). After degassing, the samples were weighed again and nitrogen (N_2) physisorption took place (using liquid N_2 with 40-point BET program). The adsorption and desorption curves were obtained for both the MOF and its electrospun analog. The BET, BJH and DFT surface area and pore diameter study were conducted, and relevant pore size distribution images were made using Prism software.

XRD: X-ray diffraction (XRD) patterns were obtained using a Malvern Panalytical Empyrean Series 3 multi-purpose X-ray diffractometer (Malvern Panalytical, Malvern, Worcestershire, UK) with Co Kα radiation and run conditions of 40 kV and 45 mA.

TGA: Thermogravimetric analysis (TGA) was performed using TA Instruments TGA 5500 (TA Instruments, New Castle, DE, USA). Samples of 9-12 mg were prepared and placed in alumina pans. Samples were heated to 1000 °C at a heating rate of 10 °C/min under compressed air atmosphere.

Microwave reactions: UIO-66 MOF was synthesized using a MARS 6 Microwave Digestion System (CEM Corporation, Matthews, NC, USA).

UV-Vis: All absorption studies related to dye removal work was performed using a Evolution 300 UV-Vis Spectrophotometer (Thermo Fisher Scientific, Waltham, MA, USA).

Dye Adsorption Experimentation: These studies were conducted similar to the presented in the previous work. [1]

Electrospinning Processing and Its Parameters

A NanoNC model ESR200R2D electrospinner was used to in the preparation of MOF integrated electrospun matrices. First, prior to ES processing, the MOF powder was milled to minimize the particle aggregation. Second, 9% PAN was dissolved in N,N-Dimethylformamide (DMF), and MOF of interest was added to the solution with a MOF to PAN weight ratio of 1:1, 1.25:1, 1.5:1, 1.75:1, and 2:1. The ideal polymer concentration should not show any bead formation at low concentrations or uneven fibers at high concentration due to increased viscosity. The mixture was magnetically stirred at 50 °C for 48 hrs to obtain a homogeneous dispersion of MOF-polymer solution.

A 6 ml syringe was used to facilitate the flow of the electrospinning solution at a constant and specific rate upon the application of a voltage. An 18-gauge needle was chosen due to its specific diameter resulting in smooth flow of the solution for all mixtures. The syringe is placed in the electrospinner at a 25° angle, spraying downward from 5.75 inches away from the rolling wire mesh.

The mixture was transferred into two 10 mL plastic syringes connected to a metal needle with a diameter of 0.8 mm. The flow rates varied between 0.1 mL/h or 0.5 mL/h and voltage from 10 kV to 30 kV. In this study, the optimum flow rate was found to be 0.5 mL/hr, and the voltage 10 kV for all material fabrication. Lower flow rates produce finer fibers with uniform morphology, while higher rates give thicker fibers or bead formation. With the application of voltage, the MOF-polymer solution was sprayed onto a stainless-steel woven wire mesh. The optimum distance between the needle and the roller was found to be 15 cm as the optimum distance. The ambient temperature and relative humidity were controlled at 23 ± 1 °C and 46 ± 3%, respectively. To stabilize the collected electrospun fiber mat and remove the solvent, the collected sample was subjected to 50°C heat in a vacuum oven for 24 hrs.

Table S1: Electrospinning parameters for different MOF-PAN solutions.

MOF	Parameters			
	Needle gauge	Solution mg	Spray angle degrees	Flow rate ml/hr
HKUST-1 (blue)	18	100	25	3
BIO MOF-1 (green)	18	100	25	3
UIO-66 (white)	18	100	25	4
MIP-202 (white)	18	100	25	7

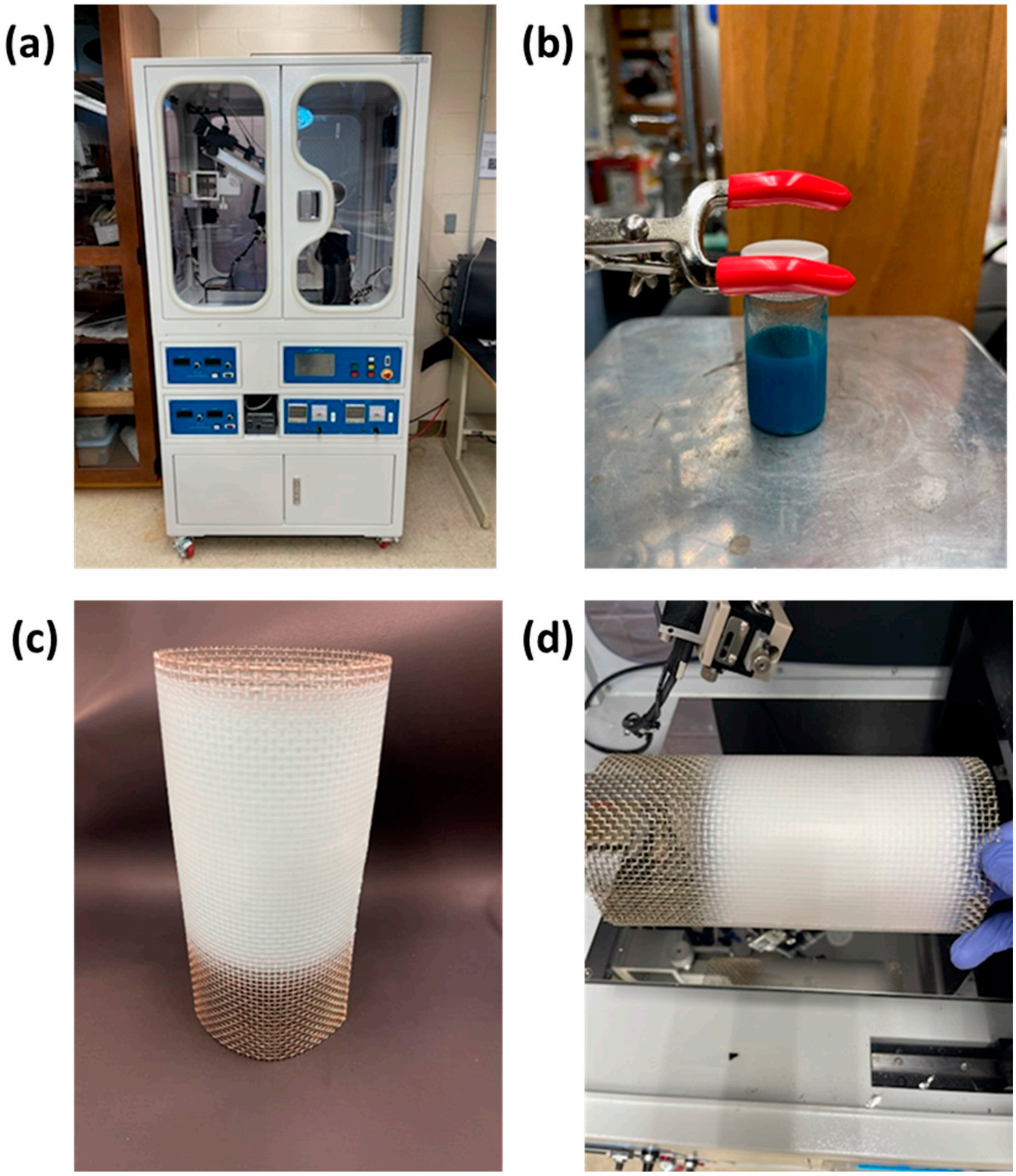

Figure S1: (a) Electrospinner, (b) stirred sample ES solution, HKUST-1 in PAN, (c) electrospun mat collected on a mesh roller, (d) collection of electrospun mat during the process.

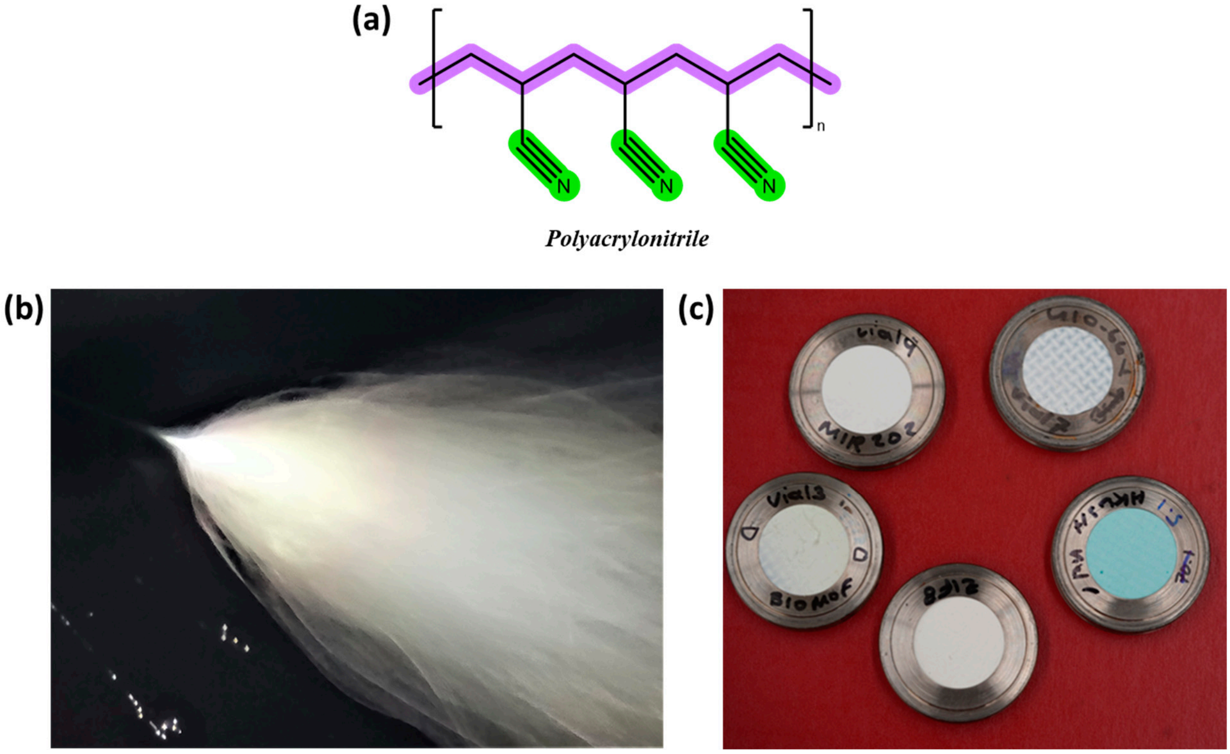

Figure S2: (a) Chemical structure, (b) MOF-PAN solution sprayed from the 18-gauge needle during the electrospinning process, and (c) XRD sample holders with electrospun MOF-PAN mat.

MOF Synthesis:

Synthesis of MIP-202

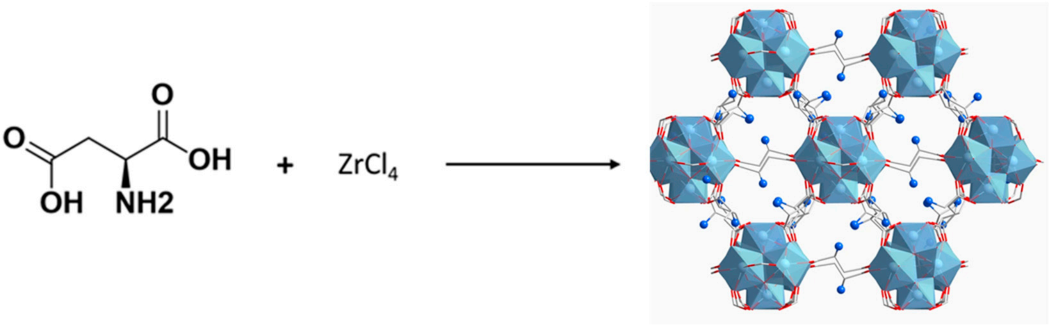

Figure S3: Synthetic scheme for the synthesis of MIP-202. The crystal structure of MIP-202(Zr) viewed along the (101) plane. [2] This figure is licensed under the Creative Commons Attribution (CC BY).

L-aspartic acid (5.59 g, 0.042 mol) was transferred into a 100 ml round bottom flask (RBF), then 20 ml of DI water was added to disperse the linker (L-asp-acid does not dissolve in DI water, therefore, to have better dispersion, a 5 min ultrasonication was followed by stirring at 400 rpm for another 5 mins). Next, $ZrCl_4$ (4.66 g, 0.020 mol) was gradually added while stirring at 400 rpm. Finally, another 20 ml of DI water was added to flush any leftover $ZrCl_4$ from the neck of the RBF. The final solution was connected to a reflux condenser and refluxed at 100 °C for 24 hrs. One reaction was refluxed with stirring at 500-600 rpm, resulting in nano-sized particles, while the other was refluxed without stirring producing micro-sized particles. The solutions obtained were transferred into falcon tubes and centrifuged at 4,000 rpm for 40 mins. The supernatant was removed, and DI water was added to the left of white solid to remove any unreacted $ZrCl_4$ and aspartic acid. The resultant material was then centrifuged at 4,000 rpm for 30 mins. The supernatant was discarded, and absolute ethanol was added to further wash the MOFs. This was again centrifuged at 4,000 rpm for 40 mins. The collected solid was subjected to vacuum for 24 hrs before ES.

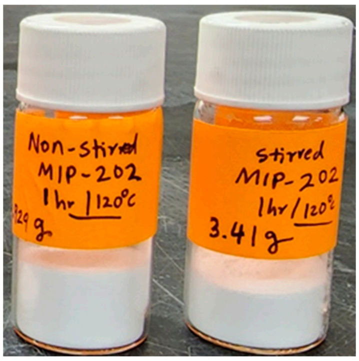

Figure S4: Physical appearance of MIP-202.

Synthesis of UIO-66 (Microwave method):

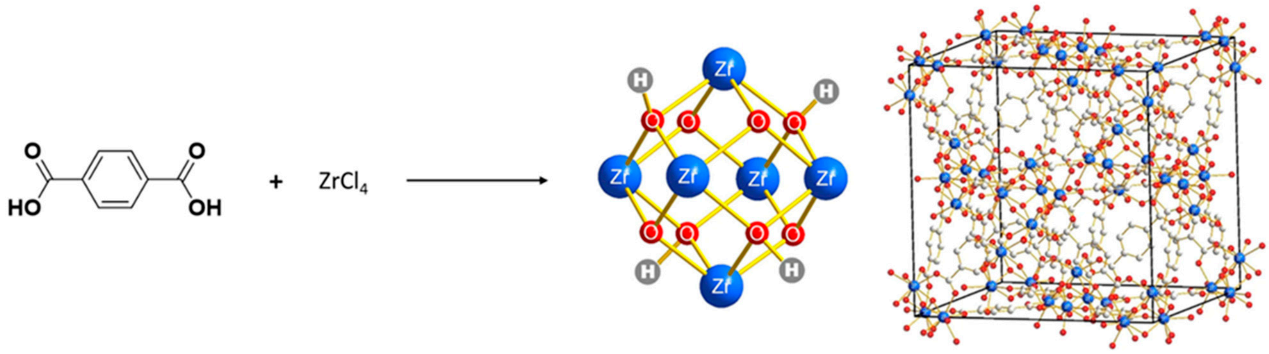

Figure S5: Synthetic scheme for synthesis of UIO-66 (unit cell image of UIO-66. [3] This figure is licensed under the Creative Commons Attribution (CC BY).

The following method was adapted from Taddei *et al*. with slight modifications.[4] A mixture solution composed of DMF (80 ml), $ZrCl_4$ (2.33 g, 10 mmol), 1,4-benzenedicarboxylic acid (1.66g, 10 mmol), acetic acid (17.1 ml, 300 mmol) and water (1.08 ml, 60 mmol) was prepared in RBF. This solution was subdivided into eight 30 ml PTFE vessels, and these were then capped and tightened using the provided caps. The vessels were placed inside CEM MARS 6™ microwave oven, and the synthesis was carried out in two stages: First, constant irradiation at 200 W was applied for 3 mins, followed with 15 mins constant irradiation at 80 W. After irradiation, vessels were immediately collected and cooled in water bath to bring them to room temperature. The obtained opaque solution was centrifuged at 4000 rpm for 2 hrs. The supernatant was decanted and fresh DMF was added, and soaked for 12 hrs, and centrifuged to remove the DMF. Then acetone was added and allowed to soak for another 12 hrs. Finally, acetone was removed by centrifugation at 4000 rpm for 30 mins. The final product was dried in an oven at 60 °C under vacuum for 24 hrs.

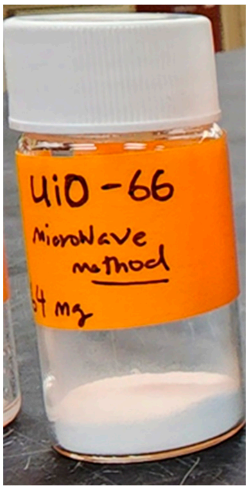

Figure S6: Physical appearance of UIO-66.

Synthesis of HKUST-1:

The following method was adapted from Chen *et al*. with slight modifications. [5] A mixture containing benzene-1,3,5-tricarboxylic acid (5.0 g, 24 mmol) and Cu (II) nitrate hemipentahydrate (10.0 g, 43 mmol) was stirred for 15 mins in 250 ml of solvent containing equal parts of 1:1:1 (83 ml each) of DMF: EtOH: DI water. This was stirred in a 500 ml pressure vessel, and once stirred, the vessel was tightly capped and placed in an oven at 85 °C for 20 hrs to yield small octahedral crystals. As for the work up, the obtained crystals were subjected to vacuum filtration using a fine filter. The obtained solid was further washed twice with 100 ml of DMF. Then, the solid/crystals were transferred into a new flask and 300 ml of DCM was added. This was kept in DCM for 3 days to remove any unreacted impurities. Each day DCM was changed with a new DCM. Finally, the solid was collected via filtration and subjected to vacuum for 24 hrs.

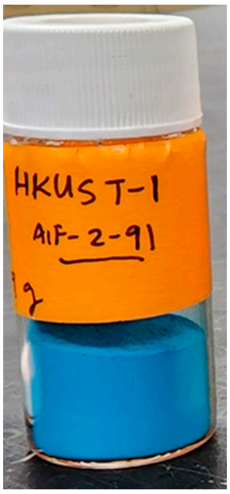

Figure S7: Physical appearance of HKUST-1.

Synthesis of BIO-MOF-1:

The following method was adapted from Yang *et al*. with slight modifications.[6] A total of 0.25 g of $CuSO_4.5H_2O$ (1mmol) was dissolved in 10 ml of DI-water. 0.225g of 6-benzylaminopurine was dissolved in 30 ml of DI water (In a 100 ml RBF), and to this 0.5 ml of HCl 36% (Conc.) was also added. This aids in protonation of amines and helps to dissolve the linker. The Cu^{2+} solution was added dropwise. Although an immediate MOF product was observed in the literature, no MOF product was observed at this stage despite multiple trials. After 24 hrs of stirring, the product was centrifuged at 4,000 rpm for 1 hr to obtain the solid product (slight green-blue product). The supernatant that was collected from this procedure was left to evaporate under ambient conditions, and that resulted in an emerald-green product. Upon centrifugation of the collected emerald-green product, it was further subjected to high vacuum/ mild elevated temperature (45 °C) resulting in a green solid. This reaction was successfully scaled up.

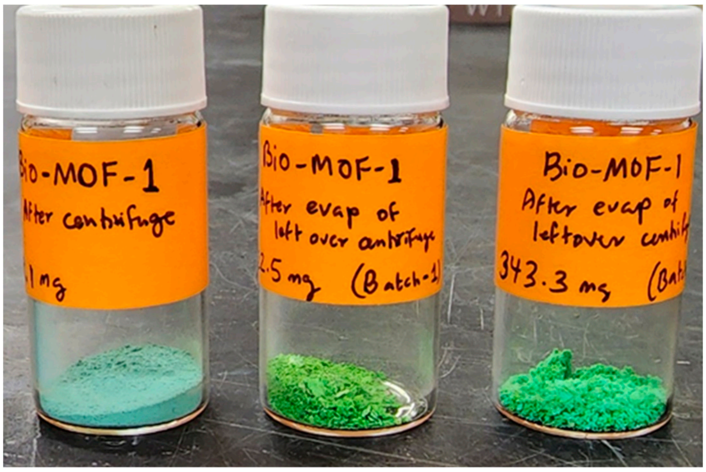

Figure S8: Physical appearance of BIO-MOF-1.

SEM/ EDX Investigation of UP-MOFs and Their PP-MOF Composites

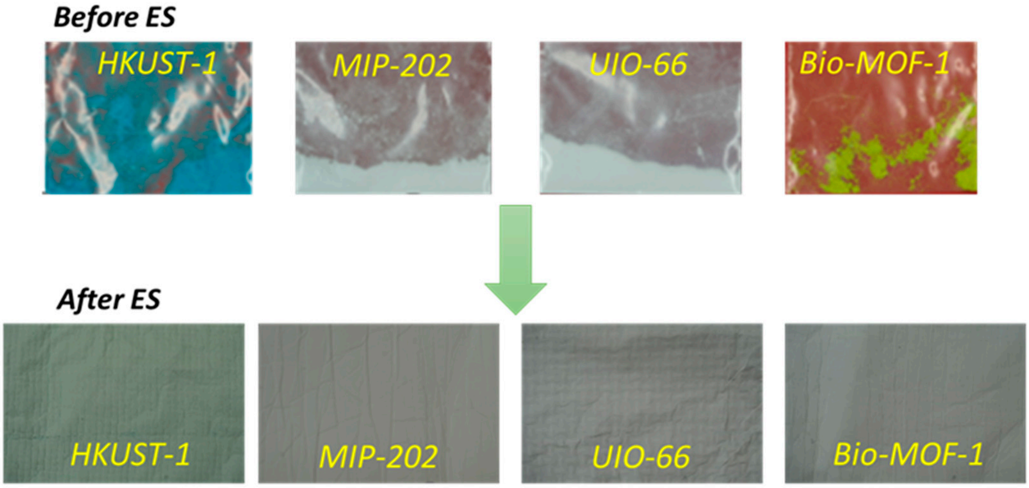

Figure S9: Pictures of HKUST-1, MIP-202, UIO-66, and BIO-MOF-1 samples before and after electrospinning.

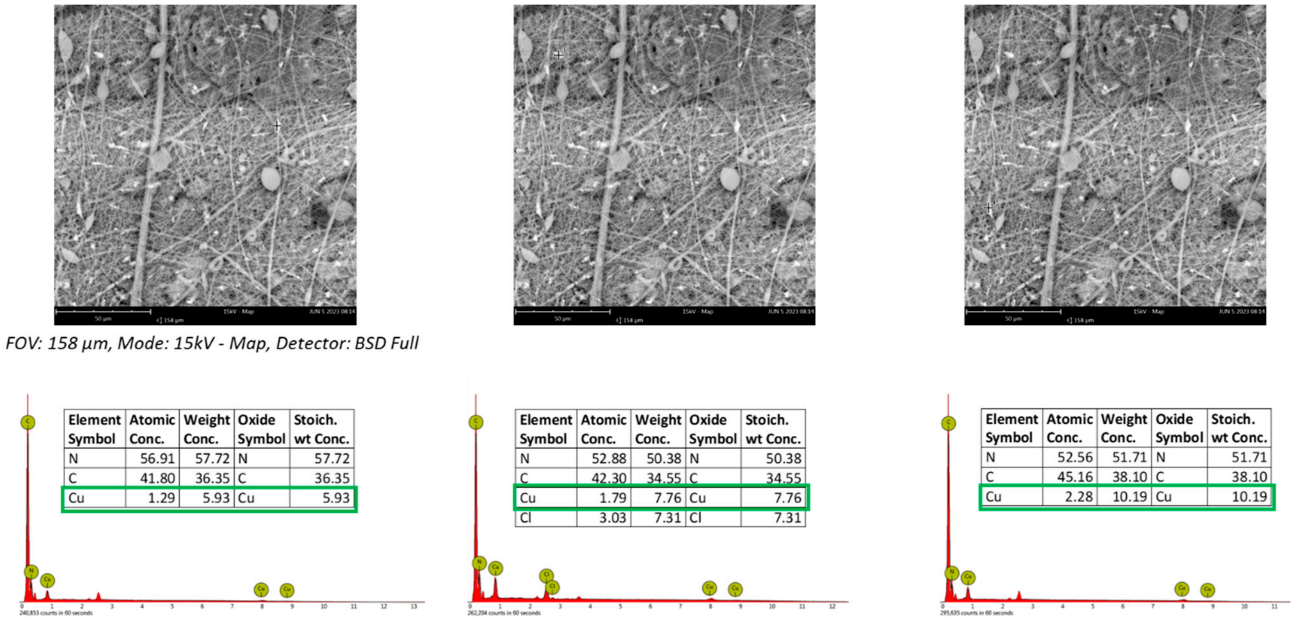

Element Symbol	Atomic Conc.	Weight Conc.	Oxide Symbol	Stoich. wt Conc.
N	56.91	57.72	N	57.72
C	41.80	36.35	C	36.35
Cu	1.29	5.93	Cu	5.93

Element Symbol	Atomic Conc.	Weight Conc.	Oxide Symbol	Stoich. wt Conc.
N	52.88	50.38	N	50.38
C	42.30	34.55	C	34.55
Cu	1.79	7.76	Cu	7.76
Cl	3.03	7.31	Cl	7.31

Element Symbol	Atomic Conc.	Weight Conc.	Oxide Symbol	Stoich. wt Conc.
N	52.56	51.71	N	51.71
C	45.16	38.10	C	38.10
Cu	2.28	10.19	Cu	10.19

Figure S10: Electrospinning BIO-MOF-1 at 10% w/w with respect to polymer mass – SEM EDX.

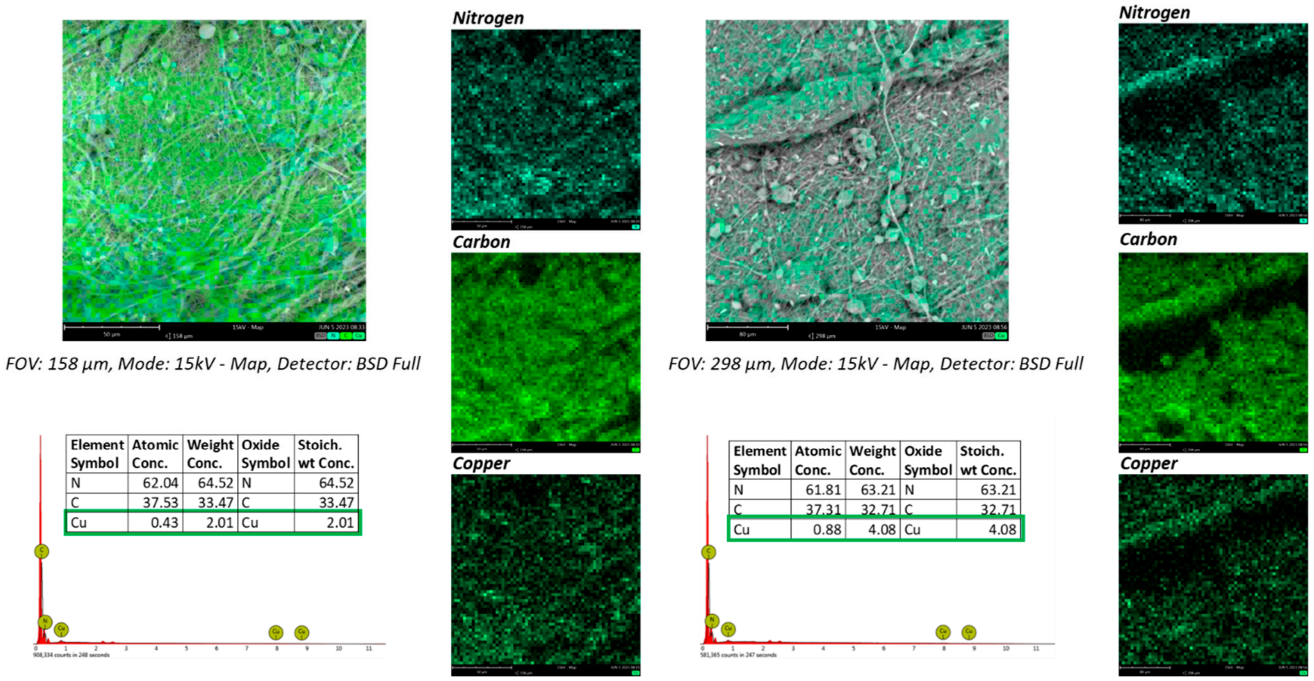

Element Symbol	Atomic Conc.	Weight Conc.	Oxide Symbol	Stoich. wt Conc.
N	62.04	64.52	N	64.52
C	37.53	33.47	C	33.47
Cu	0.43	2.01	Cu	2.01

Element Symbol	Atomic Conc.	Weight Conc.	Oxide Symbol	Stoich. wt Conc.
N	61.81	63.21	N	63.21
C	37.31	32.71	C	32.71
Cu	0.88	4.08	Cu	4.08

Figure S11: Electrospinning BIO-MOF-1 at 10% w/w with respect to polymer mass – SEM EDX (combined elemental mapping).

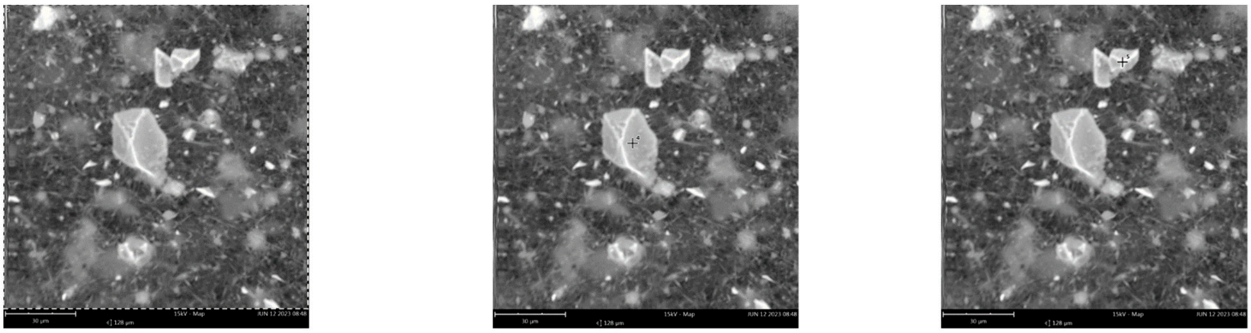

FOV: 128 µm, Mode: 15kV - Map, Detector: BSD Full

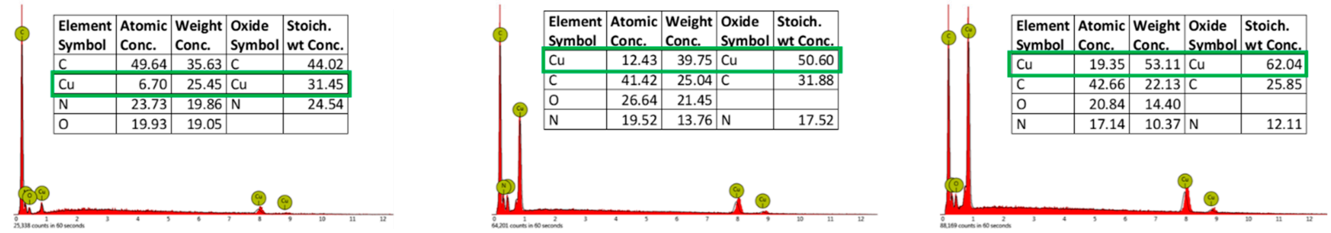

Element Symbol	Atomic Conc.	Weight Conc.	Oxide Symbol	Stoich. wt Conc.
C	49.64	35.63	C	44.02
Cu	6.70	25.45	Cu	31.45
N	23.73	19.86	N	24.54
O	19.93	19.05		

Element Symbol	Atomic Conc.	Weight Conc.	Oxide Symbol	Stoich. wt Conc.
Cu	12.43	39.75	Cu	50.60
C	41.42	25.04	C	31.88
O	26.64	21.45		
N	19.52	13.76	N	17.52

Element Symbol	Atomic Conc.	Weight Conc.	Oxide Symbol	Stoich. wt Conc.
Cu	19.35	53.11	Cu	62.04
C	42.66	22.13	C	25.85
O	20.84	14.40		
N	17.14	10.37	N	12.11

Figure S12: Electrospinning HKUST-1 at 10% w/w with respect to polymer mass – SEM EDX.

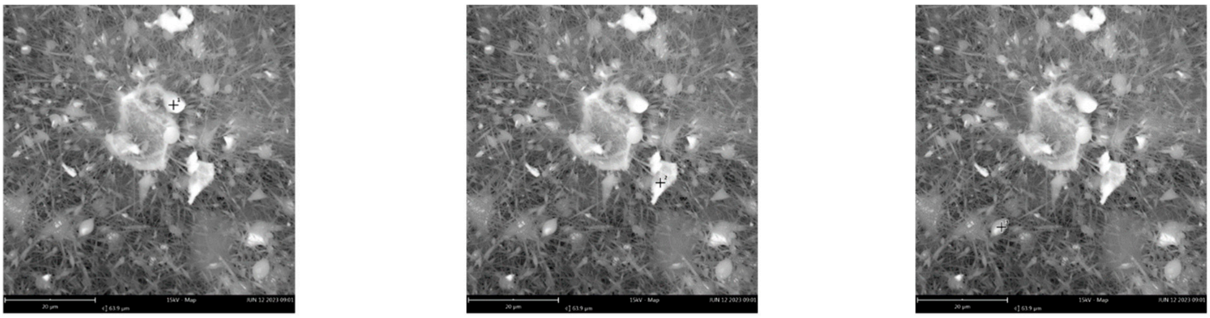

FOV: 63.9 µm, Mode: 15kV - Map, Detector: BSD Full

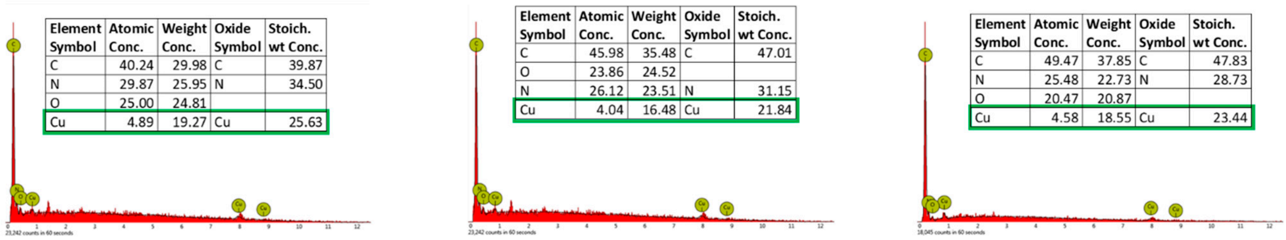

Element Symbol	Atomic Conc.	Weight Conc.	Oxide Symbol	Stoich. wt Conc.
C	40.24	29.98	C	39.87
N	29.87	25.95	N	34.50
O	25.00	24.81		
Cu	4.89	19.27	Cu	25.63

Element Symbol	Atomic Conc.	Weight Conc.	Oxide Symbol	Stoich. wt Conc.
C	45.98	35.48	C	47.01
O	23.86	24.52		
N	26.12	23.51	N	31.15
Cu	4.04	16.48	Cu	21.84

Element Symbol	Atomic Conc.	Weight Conc.	Oxide Symbol	Stoich. wt Conc.
C	49.47	37.85	C	47.83
N	25.48	22.73	N	28.73
O	20.47	20.87		
Cu	4.58	18.55	Cu	23.44

Figure S13: Electrospinning HKUST-1 at 10% w/w with respect to polymer mass – SEM EDX.

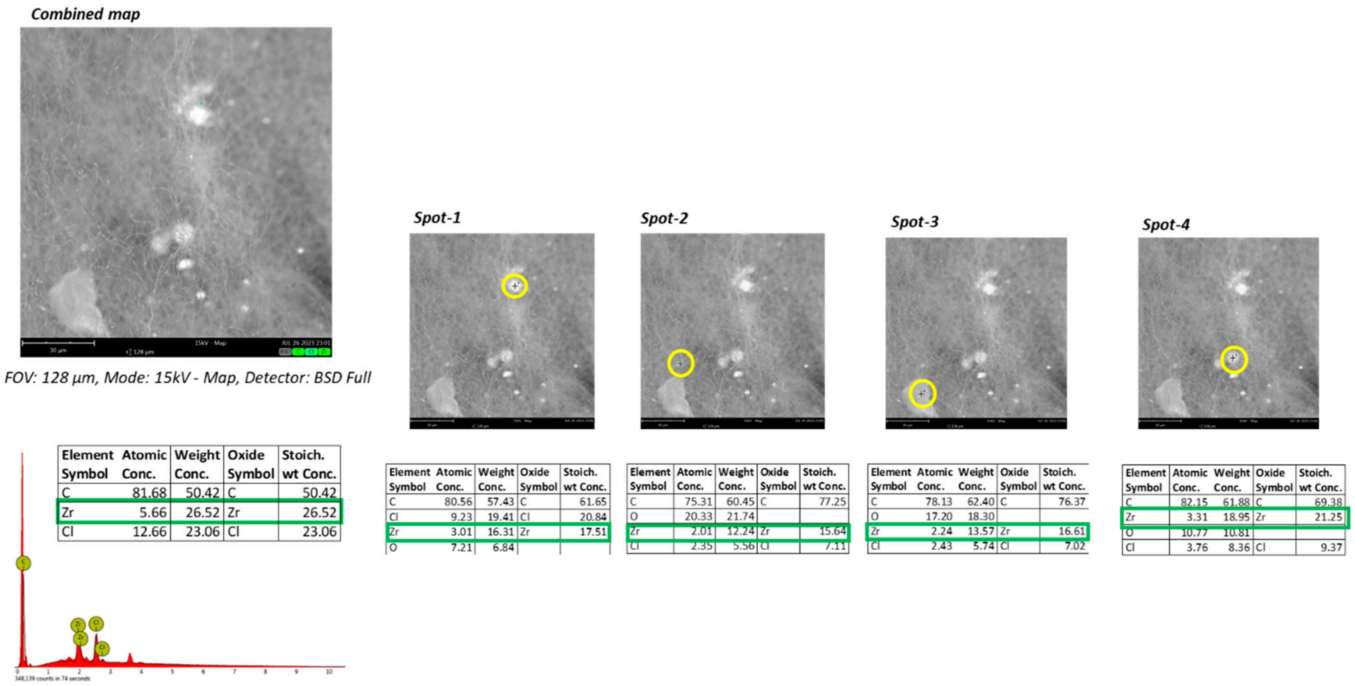

Combined map

Element Symbol	Atomic Conc.	Weight Conc.	Oxide Symbol	Stoich. wt Conc.
C	81.68	50.42	C	50.42
Zr	5.66	26.52	Zr	26.52
Cl	12.66	23.06	Cl	23.06

Spot-1

Element Symbol	Atomic Conc.	Weight Conc.	Oxide Symbol	Stoich. wt Conc.
C	80.56	57.43	C	61.65
Cl	9.23	19.41	Cl	20.84
Zr	3.01	16.31	Zr	17.51
O	7.21	6.84		

Spot-2

Element Symbol	Atomic Conc.	Weight Conc.	Oxide Symbol	Stoich. wt Conc.
C	75.31	60.45	C	77.25
O	20.33	21.74		
Zr	2.01	12.24	Zr	15.64
Cl	2.35	5.56	Cl	7.11

Spot-3

Element Symbol	Atomic Conc.	Weight Conc.	Oxide Symbol	Stoich. wt Conc.
C	78.13	62.40	C	76.37
O	17.20	18.30		
Zr	2.24	13.57	Zr	16.61
Cl	2.43	5.74	Cl	7.02

Spot-4

Element Symbol	Atomic Conc.	Weight Conc.	Oxide Symbol	Stoich. wt Conc.
C	82.15	61.88	C	69.38
Zr	3.31	18.95	Zr	21.25
O	10.77	10.81		
Cl	3.76	8.36	Cl	9.37

Figure S14: Electrospinning UIO-66 at 10% w/w with respect to polymer mass – SEM EDX.

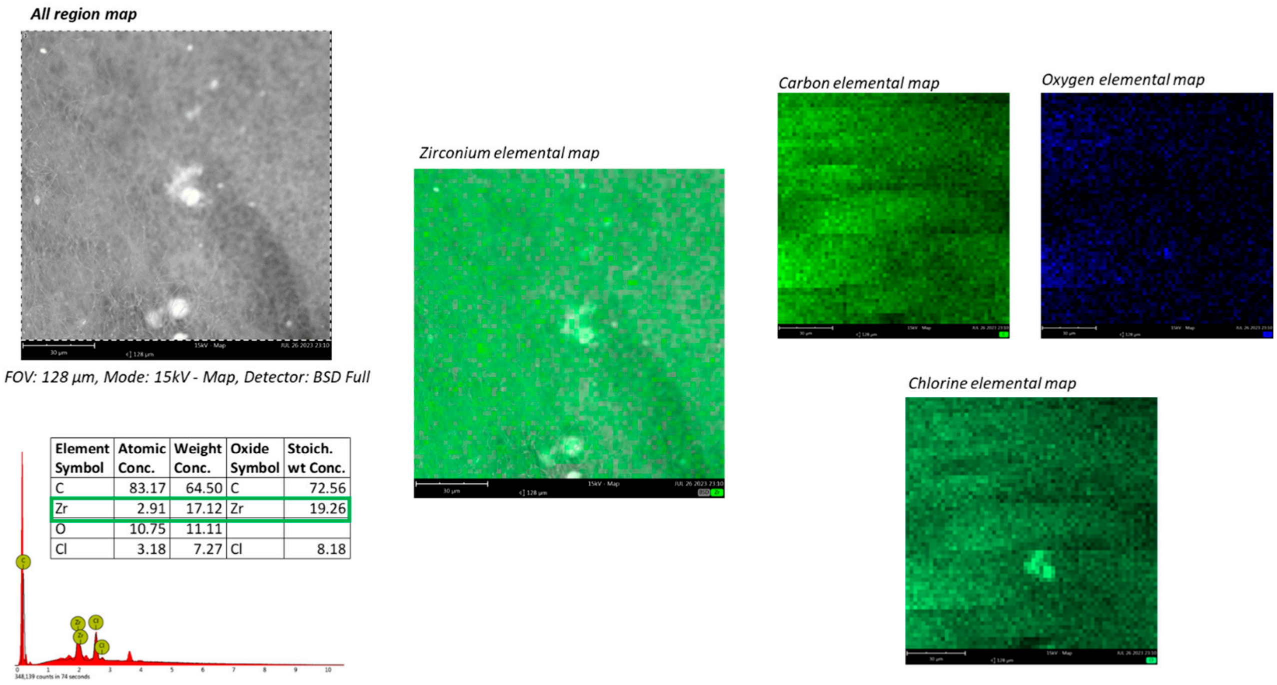

All region map

Element Symbol	Atomic Conc.	Weight Conc.	Oxide Symbol	Stoich. wt Conc.
C	83.17	64.50	C	72.56
Zr	2.91	17.12	Zr	19.26
O	10.75	11.11		
Cl	3.18	7.27	Cl	8.18

Figure S15: Electrospinning UiO-66 at 10% w/w with respect to polymer mass – SEM EDX.

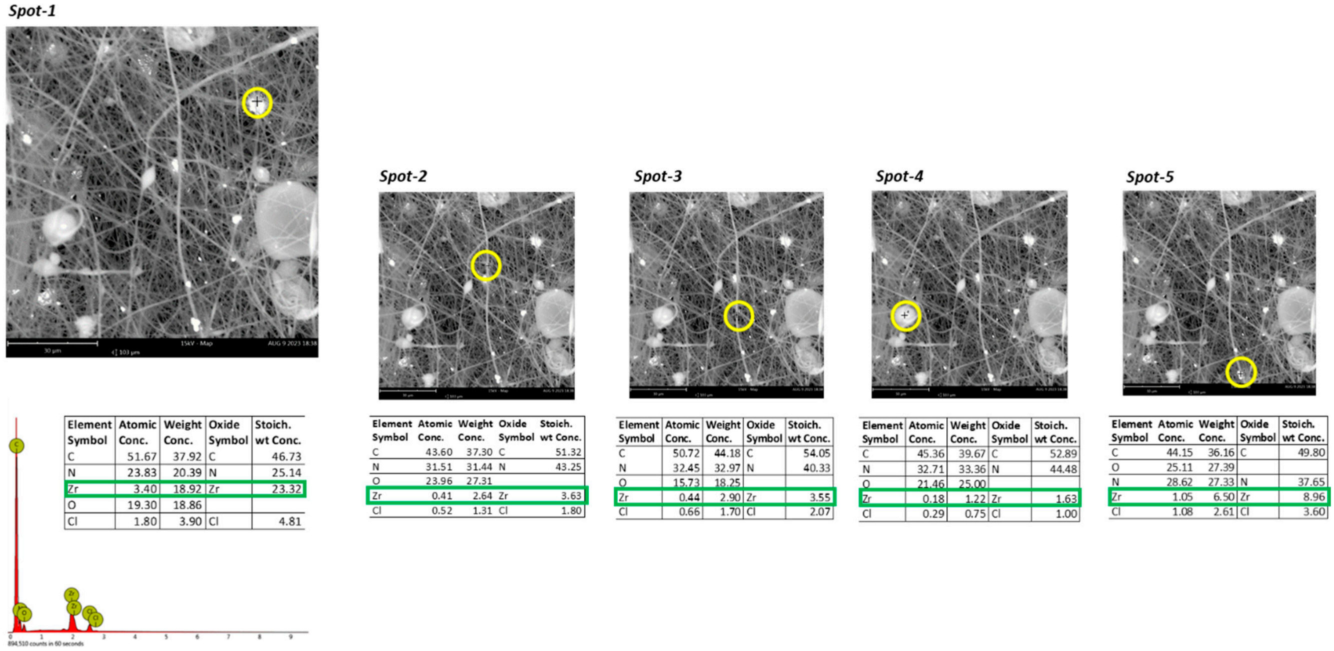

Spot-1

Element Symbol	Atomic Conc.	Weight Conc.	Oxide Symbol	Stoich. wt Conc.
C	51.67	37.92	C	46.73
N	23.83	20.39	N	25.14
Zr	3.40	18.92	Zr	23.32
O	19.30	18.86		
Cl	1.80	3.90	Cl	4.81

Spot-2

Element Symbol	Atomic Conc.	Weight Conc.	Oxide Symbol	Stoich. wt Conc.
C	43.60	37.30	C	51.32
N	31.51	31.44	N	43.25
O	23.96	27.31		
Zr	0.41	2.64	Zr	3.63
Cl	0.52	1.31	Cl	1.80

Spot-3

Element Symbol	Atomic Conc.	Weight Conc.	Oxide Symbol	Stoich. wt Conc.
C	50.72	44.18	C	54.05
N	32.45	32.97	N	40.33
O	15.73	18.25		
Zr	0.44	2.90	Zr	3.55
Cl	0.66	1.70	Cl	2.07

Spot-4

Element Symbol	Atomic Conc.	Weight Conc.	Oxide Symbol	Stoich. wt Conc.
C	45.36	39.67	C	52.89
N	32.71	33.36	N	44.48
O	21.46	25.00		
Zr	0.18	1.22	Zr	1.63
Cl	0.29	0.75	Cl	1.00

Spot-5

Element Symbol	Atomic Conc.	Weight Conc.	Oxide Symbol	Stoich. wt Conc.
C	44.15	36.16	C	49.80
O	25.11	27.39		
N	28.62	27.33	N	37.65
Zr	1.05	6.50	Zr	8.96
Cl	1.08	2.61	Cl	3.60

Figure S16: Electrospinning MIP-202 stirred 1hr at 10% w/w with respect to polymer mass – SEM EDX.

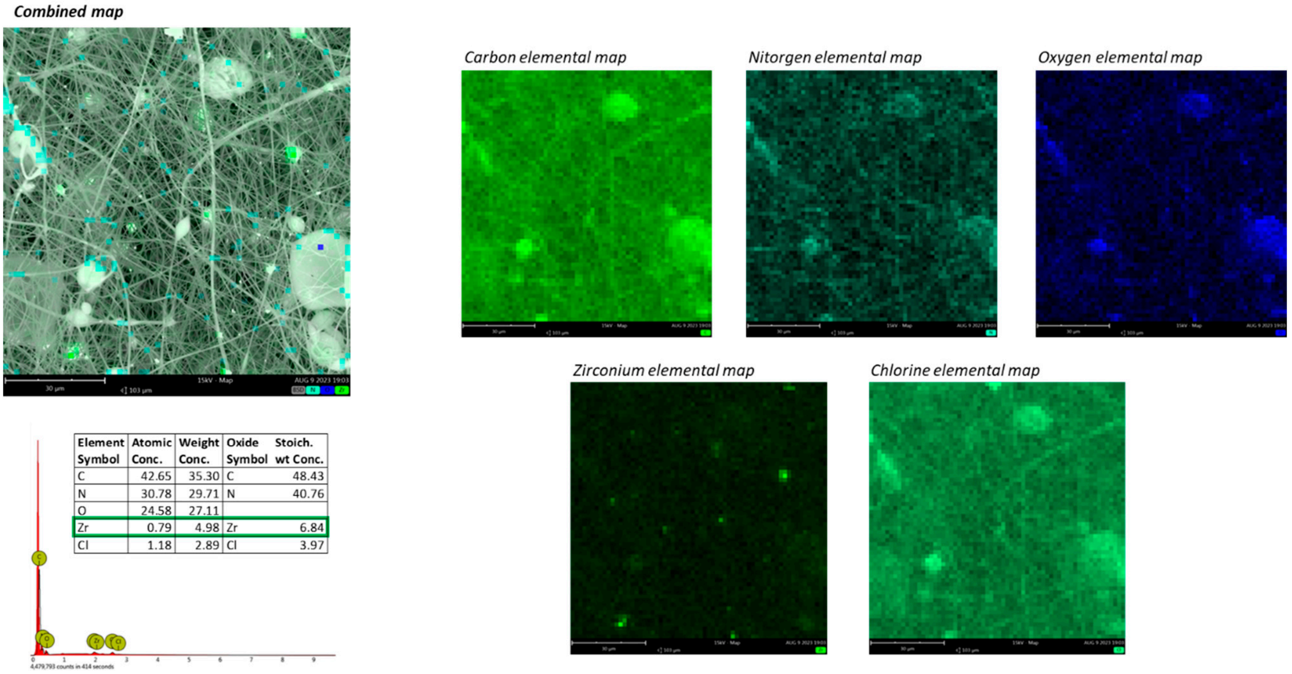

Element Symbol	Atomic Conc.	Weight Conc.	Oxide Symbol	Stoich. wt Conc.
C	42.65	35.30	C	48.43
N	30.78	29.71	N	40.76
O	24.58	27.11		
Zr	0.79	4.98	Zr	6.84
Cl	1.18	2.89	Cl	3.97

Figure S17: Electrospinning MIP-202 stirred 1hr at 10% w/w with respect to polymer mass – SEM EDX.

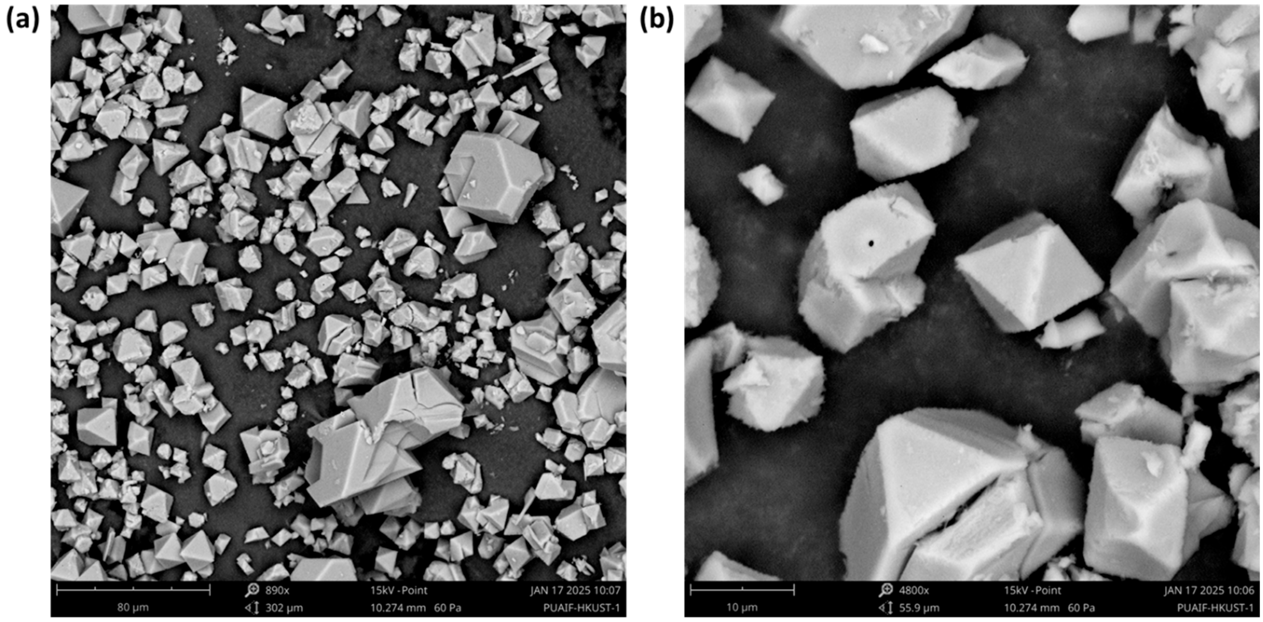

Figure S18: SEM images of HKUST-1 (a) at 890x and (b) 4800x magnification.

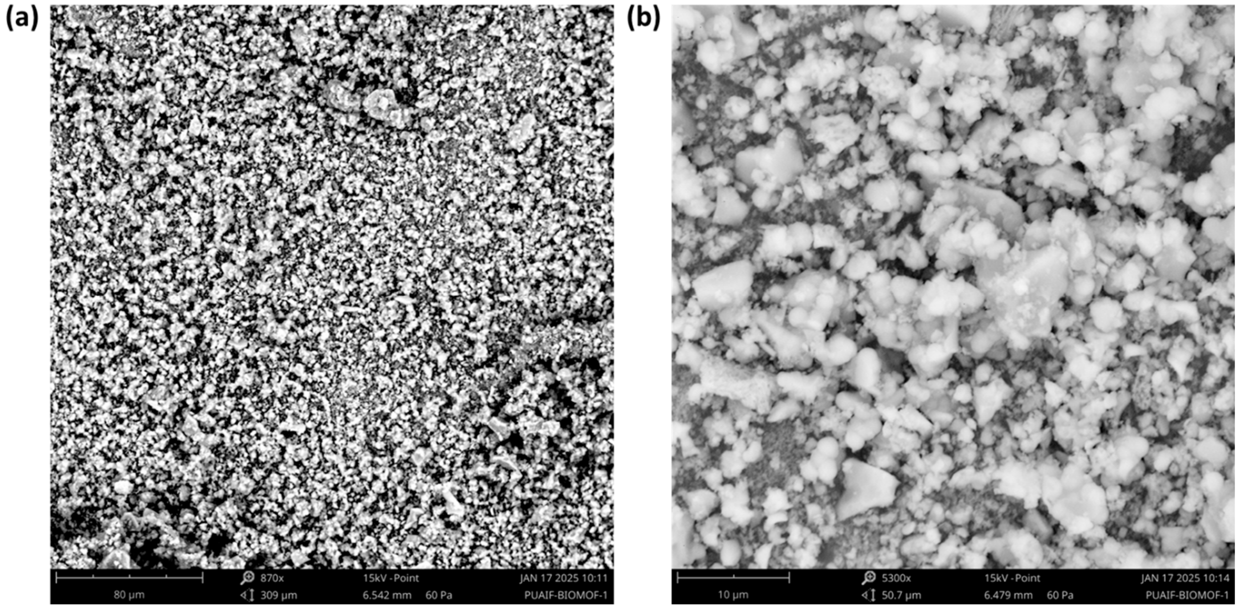

Figure S19: SEM images of BIO-MOF-1 (a) at 870x and (b) 5300x magnification.

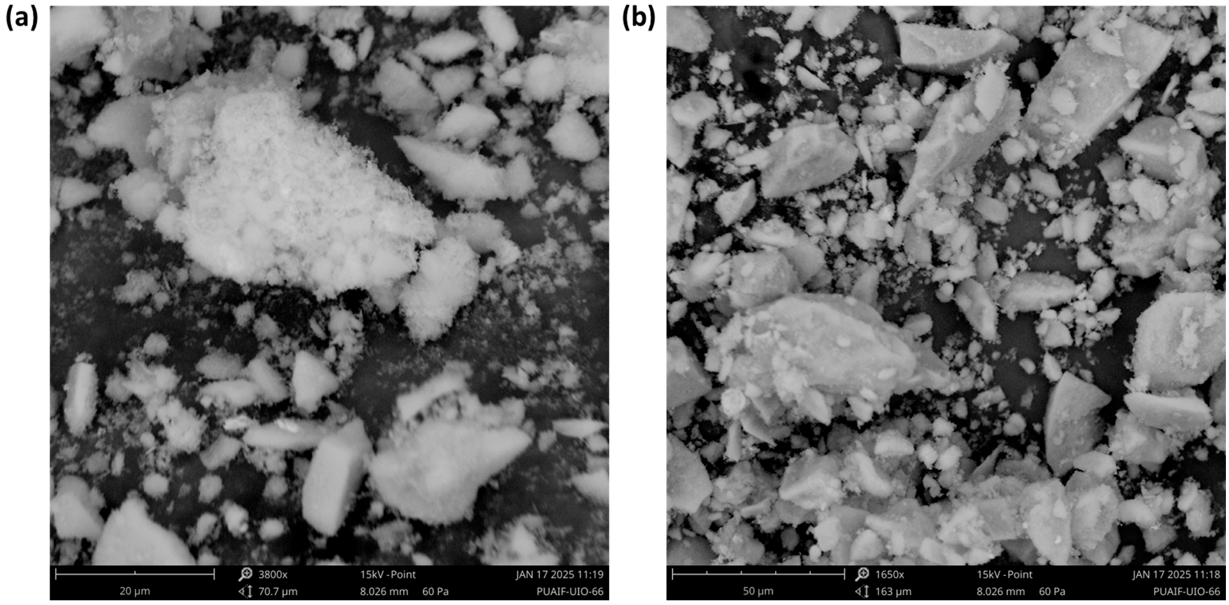

Figure S20: SEM images of UIO-66 (a) at 3800x and (b) 1650x magnification.

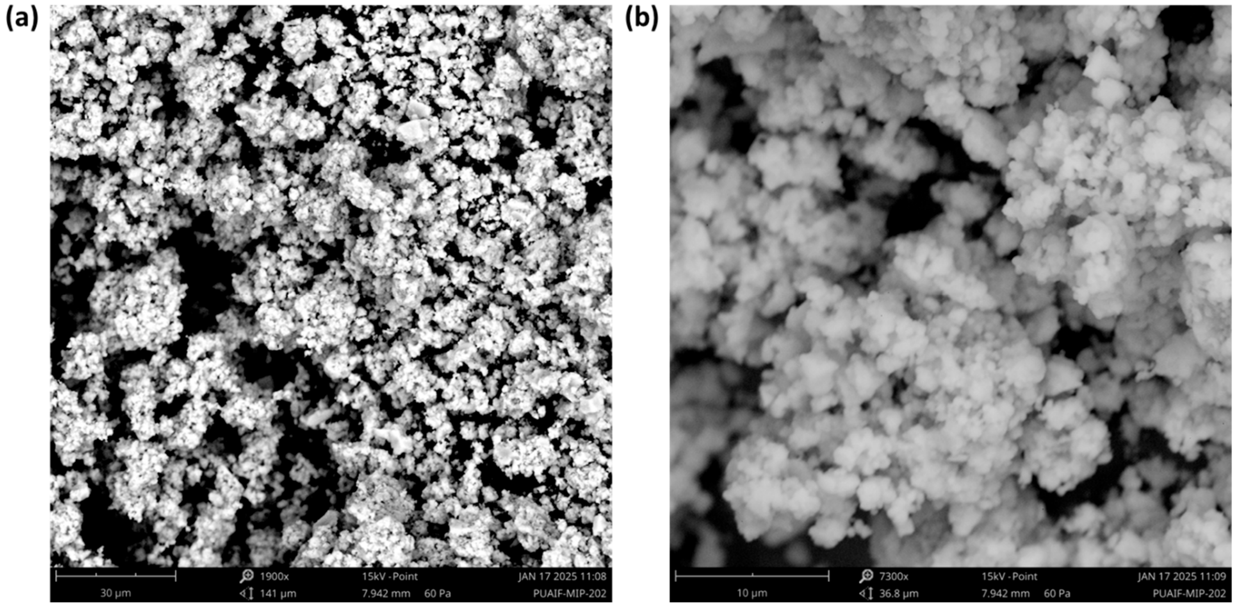

Figure S21: SEM images of UIO-66 (a) at 1900x and (b) 7300x magnification.

Thickness Percentage Calculation:

The thickness percentage was calculated using the percentage increase formula as shown:

$$\text{Percentage Increase} = \left(\frac{\text{New Diameter} - \text{Control Diameter}}{\text{Control Diameter}}\right) \times 100$$

1. **Bio MOF Espun**:
    - Control Diameter: 12.11 μm
    - Bio MOF Diameter: 17.40 μm
    - Percentage Increase: $\left(\frac{17.40-12.11}{12.11}\right) \times 100 = 43.7\%$
2. **HKUST-1 Espun**:
    - Control Diameter: 12.11 μm
    - HKUST-1 Diameter: 25.24 μm
    - Percentage Increase: $\left(\frac{25.24-12.11}{12.11}\right) \times 100 = 108.5\%$

3. **UIO-66 Espun**:
    - Control Diameter: 12.11 μm
    - UIO-66 Diameter: 19.22 μm
    - Percentage Increase: $\left(\frac{19.22-12.11}{12.11}\right) \times 100 = 58.7\%$
4. **MIP-202 Espun**:
    - Control Diameter: 12.11 μm
    - MIP-202 Diameter: 20.25 μm
    - Percentage Increase: $\left(\frac{20.25-12.11}{12.11}\right) \times 100 = 67.3\%$

Table S2: Average elemental composition of MOF-electrospun composites.

MOF	Average stoichiometric weight % of metal during spot scan	Average stoichiometric weight %of metal during full region scan Single pass
BIO-MOF-1	17.08 (3 spots)	3.05
HKUST-1	48.03 (3 spots, region-1)<br>23.64 (3 spots, region-2)	25.60
UIO-66	17.75 (4 spots)	19.26
MIP-202	8.22 (5 spots)	6.84

Table S3: BET and DFT based data fits for all materials related to N_2 physisorption.

**Sample**	**Multi-point BET surface area (m^2/g)**	**Total pore volume (cm^3/g)**	**Pore diameter (nm)**	**% Micropore volume (%) [0-9.7 nm]**	**% Mesopore volume (%) [10-40 nm]**
MIP-202	18.02	0.06	4.89	68.90	26.90
MIP-202-espun	17.96	0.06	4.88	56.53	43.47
HKUST-1	1892.97	0.35	1.38	99.98	0.02
HKUST-1 espun	468.44	0.14	1.43	98.76	1.24
UIO-66	329.91	0.20	1.63	96.37	3.62
UIO-66 espun	52.47	0.05	1.63	90.03	9.97
BIO-MOF-1	2.281	0.01	7.03	40.45	59.55
BIO-MOF-1 espun	13.98	0.04	4.89	53.07	46.93

Measuring Fiber Diameters:

The SEM image was first converted into gray scale before applying adaptive thresholding to handle the varying illumination within the image. This was followed with morphological operations to enhance the fiber structures. Finally, contours were located and average fiber diameters were measured. These steps were conducted via an open access GPT program created by Max & Kirill Dubovitsky titled "*Diagrams & Data: Research, Analyze, Visualize*." The following example shows the use of contour mapping in locating fibers.

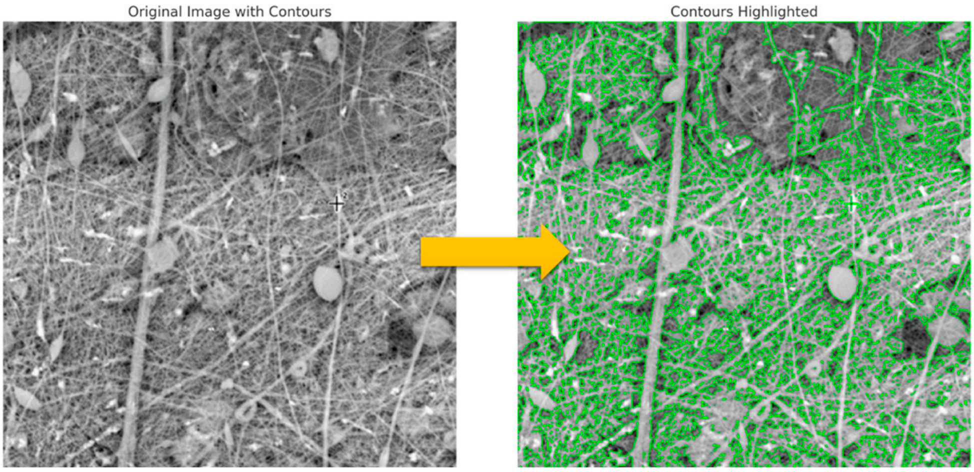

Figure S22: SEM EDX with contours.

Dye Adsorption

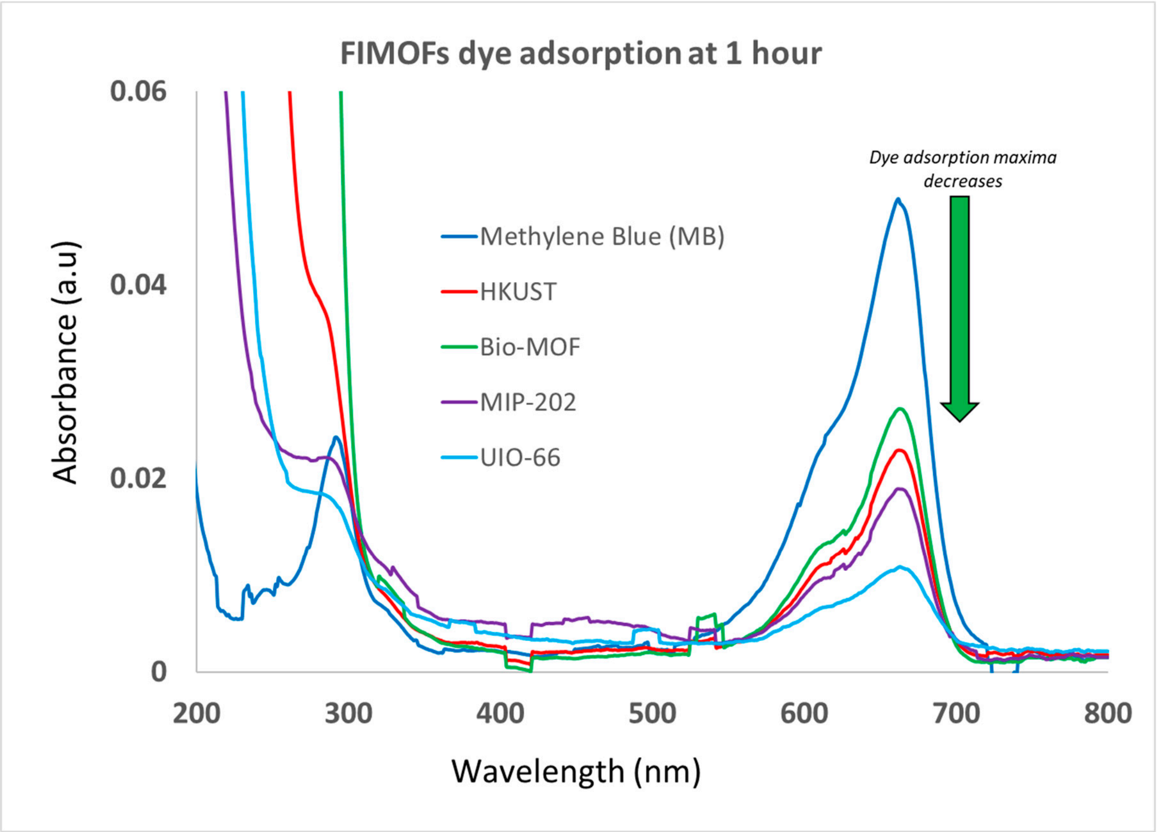

Figure S23: UV-Vis absorption spectra for dye adsorption of FIMOFs at 1-hr.

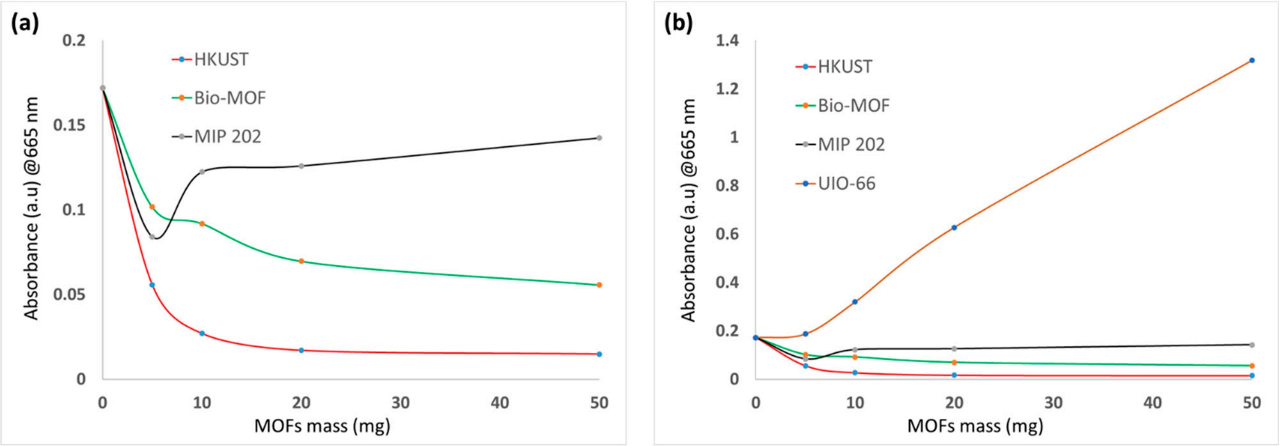

*Figure S24: Change in the absorption maximum of MB dye at 665 nm with various amounts of MOF (a) Data without UIO-66, and (b) data with UIO-66. *Plots were shown to present relatively higher absorption with UIO-66 different than the ones with other MOFs.*

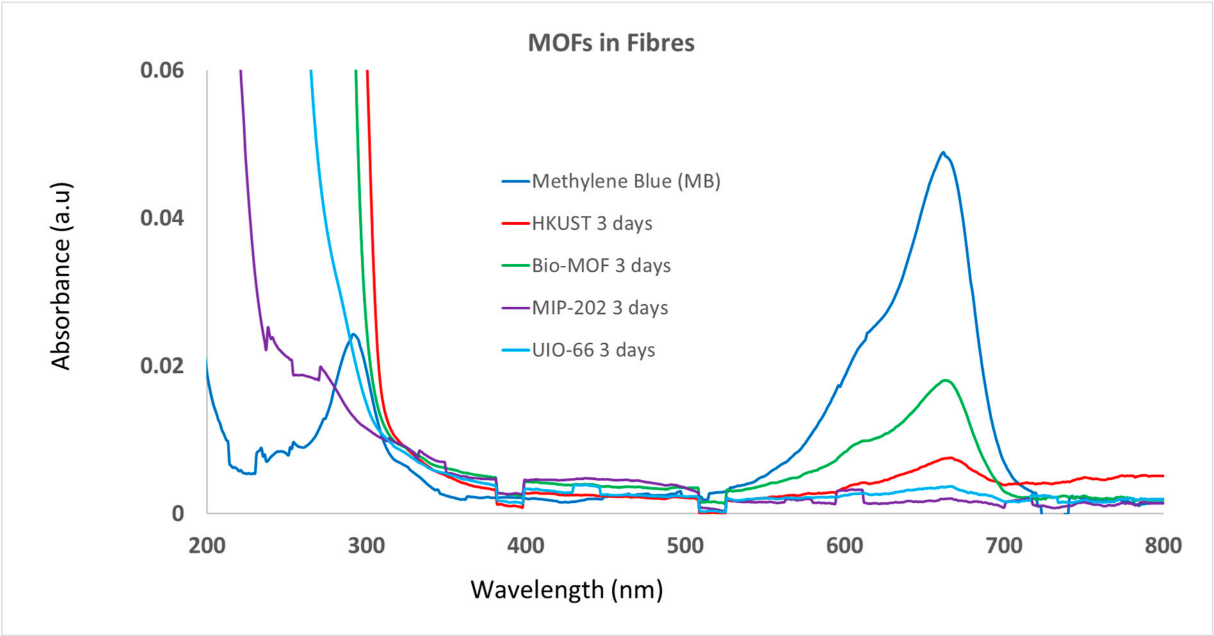

Figure S25: UV-Vis absorption spectra for FIMOFs after 3-day long dye exposure.

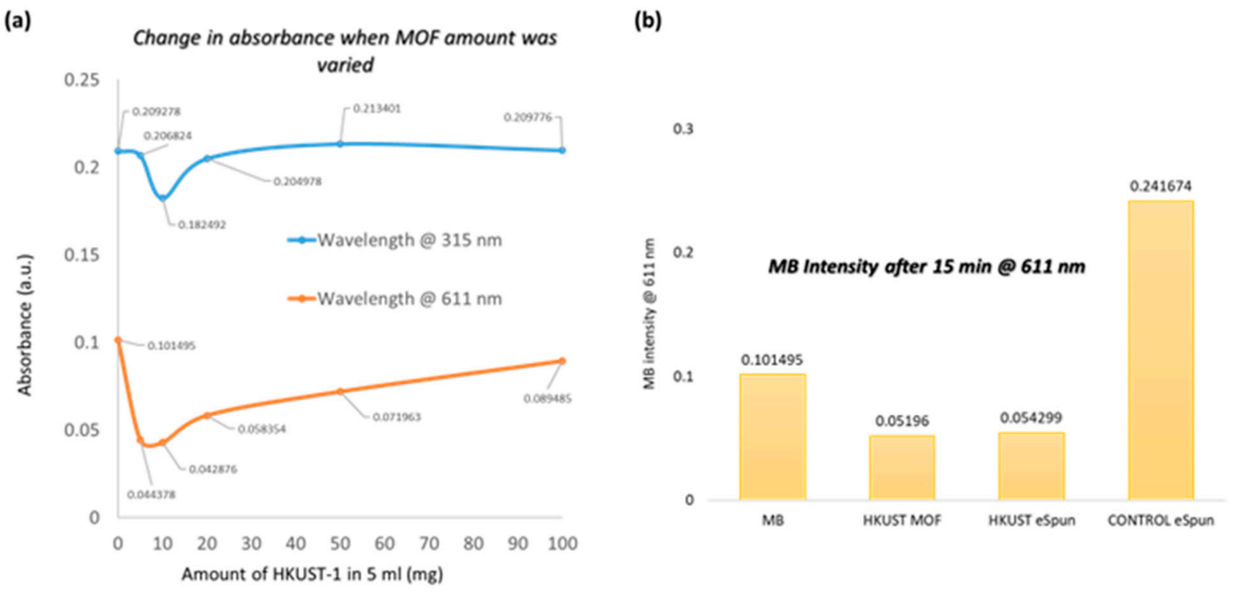

Figure S26: (a) Change in the intensity of MB dye at two absorption maxima, 315 nm (top line), and 611 nm (bottom line) with varying amount of UP-HKUST-1 powder, and (b) change in the intensity of MB dye at 611 nm for UP-HKUST-1 and PP-HKUST-1 after 1-hr exposure to dye.

Table S4: TGA analysis of UP-MOFs and PP-MOFs (FIMOF).

	MIP-202 (MOF)	*UIO-66 (MOF)*	*BioMOF-1 (MOF)*	*HKUST-1 (MOF)*	*MIP-202 (FIMOF)*	*UIO-66 (FIMOF)*	*BioMOF-1 (FIMOF)*	*HKUST-1 (FIMOF)*
Initial weight (mg)	10.633	11.944	11.868	10.311	11.738	10.633	N/A	9.180
Final weight @ 1000C (mg)	3.350	3.605	2.949	2.681	0.406	3.350	N/A	3.092
Content wt/wt%	**31.5**	**30.2**	**24.8**	**26.0**	**3.5**	**31.5**	**N/A**	**33.7**

References

1. Diab, K.E.; Salama, E.; Hassan, H.S.; Abd El-moneim, A.; Elkady, M.F. Biocompatible MIP-202 Zr-MOF Tunable Sorbent for Cost-Effective Decontamination of Anionic and Cationic Pollutants from Waste Solutions. *Sci Rep* **2021**, *11*, 6619, doi:10.1038/s41598-021-86140-2.
2. Wang, S.; Wahiduzzaman, M.; Davis, L.; Tissot, A.; Shepard, W.; Marrot, J.; Martineau-Corcos, C.; Hamdane, D.; Maurin, G.; Devautour-Vinot, S.; et al. A Robust Zirconium Amino Acid Metal-Organic Framework for Proton Conduction. *Nature Communications* **2018**, *9*, 4937, doi:10.1038/s41467-018-07414-4.
3. Gu, Q.; Ng, H.Y.; Zhao, D.; Wang, J. Metal–Organic Frameworks (MOFs)-Boosted Filtration Membrane Technology for Water Sustainability. *APL Materials* **2020**, *8*, 040902, doi:10.1063/5.0002905.
4. Taddei, M.; Dau, P.V.; Cohen, S.M.; Ranocchiari, M.; Van Bokhoven, J.A.; Costantino, F.; Sabatini, S.; Vivani, R. Efficient Microwave Assisted Synthesis of Metal–Organic Framework UiO-66: Optimization and Scale Up. *Dalton Trans.* **2015**, *44*, 14019–14026, doi:10.1039/C5DT01838B.
5. Chen, Y.; Mu, X.; Lester, E.; Wu, T. High Efficiency Synthesis of HKUST-1 under Mild Conditions with High BET Surface Area and CO2 Uptake Capacity. *Progress in Natural Science: Materials International* **2018**, *28*, 584–589, doi:10.1016/j.pnsc.2018.08.002.
6. Yang, Y.; Gong, Y.; Li, X.; Li, M.; Wei, Q.; Zhou, B.; Zhang, J. Alkaline-Stable Peroxidase Mimics Based on Biological Metal–Organic Frameworks for Recyclable Scavenging of Hydrogen Peroxide and Detecting Glucose in Apple Fruits. *ACS Sustainable Chem. Eng.* **2022**, *10*, 10685–10698, doi:10.1021/acssuschemeng.2c03184.
